# Bioactivity and Neuroprotective Effects of Extra Virgin Olive Oil in a Mouse Model of Cerebral Ischemia: An In Vitro and In Vivo Study

**DOI:** 10.3390/ijms26041771

**Published:** 2025-02-19

**Authors:** Salvatore Scacco, Silvia Acquaviva, Fábio França Vieira e Silva, John H. Zhang, Lorenzo Lo Muzio, Gaetano Corso, Vito Carlo Alberto Caponio, Pierluigi Reveglia, Lucia Lecce, Maria Eleonora Bizzoca, Prativa Sherchan, Stefania Cantore, Andrea Ballini

**Affiliations:** 1 Clinical Biochemistry Unit, Department of Translational Biomedicine and Neuroscience-DiBraiN, School of Medicine, University of Bari “Aldo Moro”, 70124 Bari, Italy; salvatore.scacco@uniba.it (S.S.); silvia.acquaviva@uniba.it (S.A.); 2 Department of Precision Medicine, University of Campania Luigi Vanvitelli, Via De Crecchio, 7, 80138 Naples, Italy; fabio.francavieiraesilva@unicampania.it; 3 Department of Physiology and Pharmacology, Loma Linda University, Loma Linda, CA 92350, USA; jhzhang@llu.edu; 4 Department of Anesthesiology, Loma Linda University, Loma Linda, CA 92350, USA; 5 Department of Neurosurgery, Loma Linda University, Loma Linda, CA 92350, USA; psherchan@llu.edu; 6 Department of Clinical and Experimental Medicine, University of Foggia, 71100 Foggia, Italy; lorenzo.lomuzio@unifg.it (L.L.M.); gaetano.corso@unifg.it (G.C.); vitocarlo.caponio@unifg.it (V.C.A.C.); pierluigi.reveglia@unifg.it (P.R.); lucia.lecce@unifg.it (L.L.); mariaeleonora.bizzoca@unifg.it (M.E.B.); andrea.ballini@unifg.it (A.B.)

**Keywords:** cerebral ischemia, mitochondria homeostasis, polyunsaturated fatty acids

## Abstract

Cerebral ischemia is a pathological condition characterized by complete blood and oxygen supply deprivation to neuronal tissue. The ischemic brain compensates for the rapid decline in ATP levels by increasing the anaerobic glycolysis rate, which leads to lactate accumulation and subsequent acidosis. Astrocytes play a critical role in regulating cerebral energy metabolism. Mitochondria are significant targets in hypoxia-ischemia injury, and disruptions in mitochondrial homeostasis and cellular energetics worsen outcomes, especially in the elderly. Elevated levels of n-3 polyunsaturated fatty acids (PUFAs) protect the adult and neonatal brain from ischemic damage by suppressing inflammation, countering oxidative stress, supporting neurovascular unit reconstruction, and promoting oligodendrogenesis. This study examines extra virgin olive oil (EVOO) treatment on TNC WT and TNC M23 cells, focusing on oxygen consumption and reactive oxygen species (ROS) production. This study investigates the effects of different durations of middle cerebral artery occlusion (MCAo) and EVOO administration on cerebral infarct volume, neurological scores, mitochondrial function, and cell viability. Cerebral infarct volume increased with longer ischemia times, while EVOO treatment (0.5 mg/kg/day) significantly reduced infarction across all MCAo durations. The oxygen consumption assays demonstrate EVOO’s dose-dependent stimulation of mitochondrial respiration in astrocytes, particularly at lower concentrations. Furthermore, EVOO-treated cells reduce ROS production during hypoxia, improve cell viability under ischemic stress, and enhance ATP production in ischemic conditions, underscoring EVOO’s neuroprotective potential.

## 1. Introduction

Cerebral ischemia occurs when the blood and oxygen supply to the brain is reduced or completely blocked, leading to neuronal damage and impaired brain function [1]. It can present in two forms: focal ischemia, which is localized and often caused by blood clots, and global ischemia, where blood flow is diminished or halted across multiple brain regions. A key mechanism of ischemic injury is the oxidative phosphorylation disruption, which produces adenosine triphosphate (ATP) in cells [2]. When blood flow is restricted, ATP production drops, causing a shift to anaerobic glycolysis, which leads to lactate buildup and acidosis, further worsening cellular damage [1,3].

Ischemia/reperfusion (I/R) injury occurs when blood flow is restored after an ischemia period [4]. Although reperfusion is necessary, it paradoxically generates reactive oxygen species (ROS) and reactive nitrogen species (RNS), which exacerbate cell damage [5,6,7]. During reperfusion, the oxygen reintroduction leads to the excessive production of free radicals, causing oxidative stress and inflammation, which can generate neuronal death and damage. Glutamate excitotoxicity, a process where excessive glutamate release leads to calcium influx and neuronal injury, is also a significant mechanism of damage in ischemia [8].

Astrocytes, the primary glial cells in the brain, mitigate ischemic damage by maintaining cerebral homeostasis, regulating neurotransmitters like glutamate, and controlling blood flow [9,10,11]. During ischemic events, astrocytes help buffer excitotoxicity by taking up excess glutamate and converting it to glutamine [12,13]. However, during I/R, astrocytic functions can become overwhelmed, contributing to further oxidative stress and inflammation [14,15].

Mitochondria are crucial in energy production and ROS generation, particularly in the ischemic injury context. ROS utilize the electron transport chain (ETC) to convert electron donors, such as reduced nicotinamide adenine dinucleotide (NADH), into bioavailable chemical energy [16]. This process involves a series of oxidation/reduction reactions that transfer electrons, facilitating proton transport across the inner mitochondrial membrane. The resultant electrochemical gradient drives ATP synthesis. The ETC comprises five enzyme complexes: NADH-CoQ reductase (Complex I), Succinate-CoQ reductase (Complex II), CoQ-cytochrome c reductase (Complex III), cytochrome c oxidase (Complex IV), and ATP synthase (Complex V), all of which are integral membrane proteins [16,17].

Mitochondrial Complex I initiates the electron transport chain by oxidizing NADH, which releases two electrons that are transferred through the flavin mononucleotide (FMN) prosthetic group and nine iron-sulfur (FeS) clusters to coenzyme Q (CoQ). This reduction process also generates superoxide, a type of ROS, through the transfer of protons that contribute to the proton motive force necessary for ATP synthesis. Notably, the Complex I inhibition by substances like Rotenone can increase superoxide production, underscoring its role in oxidative stress [17].

In addition to Complex I, Complexes II, III, and IV are essential for maintaining mitochondrial function, especially during ischemic events. Complex II, or Succinate-Q oxidoreductase, links the citric acid cycle to the ETC by catalyzing the oxidation of succinate to fumarate, facilitating electron transfer to CoQ. Complex III, known as Q-cytochrome c reductase, serves as a significant site for ROS production and utilizes a dimer structure to drive the Q cycle, releasing protons into the intermembrane space [18,19]. Complex IV, or Cytochrome C oxidase, is crucial for transferring electrons to molecular oxygen, leading to the production of water and further contributing to the electrochemical gradient. ATP synthase (Complex V) then harnesses this gradient to synthesize ATP, making it pivotal for cellular energy metabolism. The overall mitochondrial function efficiency declines with age, heightening the brain’s vulnerability to ischemic damage, thus highlighting the importance of targeting oxidative phosphorylation and mitochondrial pathways for neuroprotection [20,21].

Polyunsaturated fatty acids (PUFAs) are essential dietary components characterized by one or more double bonds in their chemical structure. PUFAs are categorized into two main groups: omega-3 (n-3) and omega-6 (n-6) fatty acids. Within the brain, docosahexaenoic acid (DHA), a vital n-3 PUFA, plays a critical role in neuronal membranes and regulates key neuronal functions such as ion channel interactions and neurotransmitter release [22]. The neurotrophic properties of n-3 PUFAs are well-documented, highlighting their significant impact on neurodevelopment [23,24].

**Figure 1:**
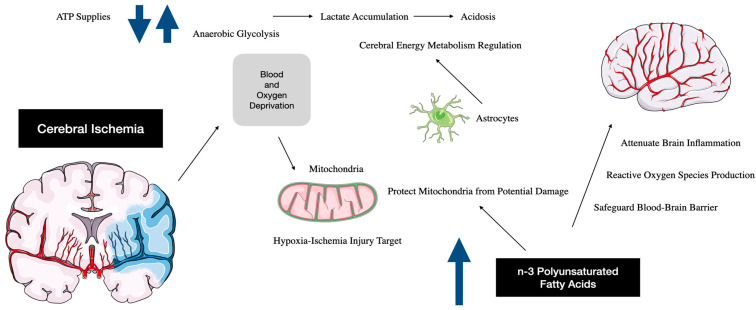
Dietary omega-3 polyunsaturated fatty acids (n-3 PUFAs) have been shown to protect the neonatal brain against hypoxic/ischemic (H/I) injury, enhancing pro-survival signaling by promoting the biosynthesis of protector factors in neuronal cell membranes. The flowchart was designed using MedicalArt software, https://www.medicalart.co.uk/ (Paintworks, Bristol, UK).

In addition to their role in neurodevelopment, elevated n-3 PUFA levels confer protective benefits to adult and neonatal brains against ischemic damage. These protective mechanisms include inflammatory response suppression, oxidative stress mitigation, neurovascular unit reconstruction support, and oligodendrogenesis promotion. It is increasingly recognized that these beneficial effects extend beyond the direct influence of DHA alone [22]. The broader context of these protective effects includes several extra virgin olive oil (EVOO) components, such as tocopherols, sterols, polyphenols, pigments, and other minor constituents within its unsaponifiable fraction [25] (Figure 1).

A promising research area focuses on the protective effects of dietary components, like EVOO, in ischemia models. EVOO is rich in bioactive compounds, including polyphenols, tocopherols, and sterols, which are known for their antioxidant and anti-inflammatory properties [26]. Studies have shown that polyphenols in EVOO, such as hydroxytyrosol, can reduce oxidative stress and inflammation, both key contributors to ischemic damage. These compounds may also enhance mitochondrial function and reduce ROS production during reperfusion, thus protecting brain tissue from further injury [27,28,29,30,31].

Despite the knowledge of its protective effects in ischemia models, many questions remain unanswered. While EVOO’s antioxidant properties have been well-documented, its specific mechanisms in preventing or reducing ischemic injury in the brain need further exploration. Additionally, it is unclear how the bioactive components in EVOO interact with key pathways involved in oxidative phosphorylation and mitochondrial dysfunction during ischemic events. This study aims to investigate EVOO’s protective effects in ischemic injury, focusing on mitochondrial function, oxidative stress, and astrocyte activity. We hypothesize that EVOO’s bioactive components enhance mitochondrial resilience, reduce oxidative stress, and protect astrocyte function during ischemia and reperfusion. Using in vitro and in vivo models, we will explore mitochondrial respiration, oxidative stress, and astrocytic responses under normal conditions and ischemic stress, examining neurobehavioral outcomes and cerebral infarct volume in treated rats. Our goal is to provide insights into EVOO’s therapeutic potential in mitigating ischemic stroke damage.

## 2. Results

### 2.1. EVOO Chemical Composition

**Figure 2:**
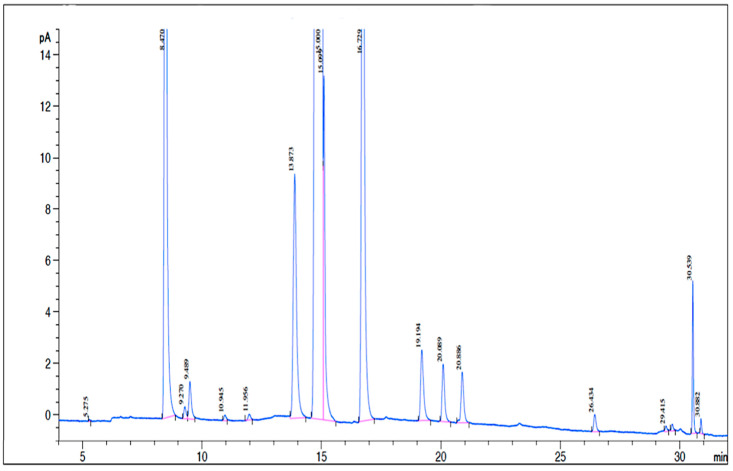
Analysis of EVOO methyl esters of fatty acid isomers by gas chromatography with a flame ionization detector (GC-FID).

The comprehensive chemical composition of fatty acids and polyphenols presented in EVOO (‘Tesserae’ Olio EVO, Frantoio Famiglia Di Palma, Canosa di Puglia, Italy) and used for the experiments are reported in Table 1 and Table 2, respectively. Moreover, a chromatogram reporting the retention time of the fatty acids detected is reported in Figure 2.

### 2.2. Cerebral Infarct Volume Evaluation

**Figure 3:**
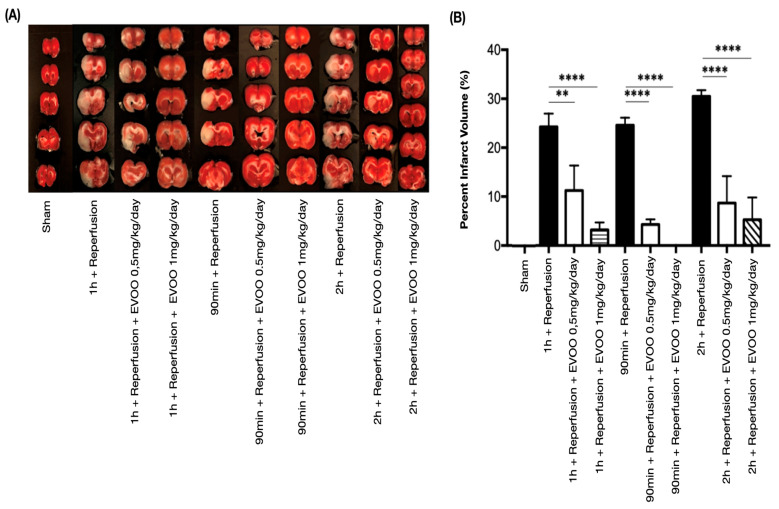
Cerebral infarct volume assessment. The infarct area and ipsilateral hemisphere of each brain slice were calculated via ImageJ (ImageJ 1.4; NIH, Bethesda, MD, USA), yielding a corrected infarct volume ratio relative to the total contralateral hemisphere volume. (**A**) Images show 2 mm rat brain slices incubated in 2% 2,3,5-Triphenyltetrazolium chloride (TTC) for 10 min, and then replaced with 1% phosphate-buffered saline (PBS) for 10 min and photographed. The white area on the brain slices indicates infarct formation. (**B**) Extra-virgin olive oil (EVOO) effects on ischemia induced by middle cerebral artery occlusion (MCAo) in rats. Treatment with EVOO was administered via stomach tube gavage for 24 h at varying concentrations (0.5 mg/kg/day and 1 mg/kg/day) and different MCAo durations (1 h, 90 min, and 2 h with 24-h reperfusion). A statistically significant reduction in infarct volume is observed in EVOO-treated rats, with *p*-values indicating significance as follows: ** *p* < 0.01; **** *p* < 0.0001.

Upon evaluating cerebral infarct volume, it is evident that brains subjected to ischemia for 1 h, 90 min, and 2 h, with 24 h of reperfusion, display progressively larger infarct volumes corresponding to middle cerebral artery occlusion (MCAo) duration. This observation underscores the direct correlation between the ischemic insult extent and resultant brain tissue damage. Interestingly, treatment with 0.5 mg/kg/day of EVOO showed a notable and statistically significant reduction in infarction volume compared to untreated controls. This beneficial effect was consistent across all three MCAo durations studied (1 h, 90 min, and 2 h), mirroring the protective efficacy observed with the higher dose of 1 mg/kg/day. These findings suggest that EVOO treatment has a dose-dependent neuroprotective role in mitigating ischemia-induced brain injury, highlighting its potential therapeutic value in ischemic stroke (Figure 3).

### 2.3. Neurological Score Evaluation

Upon assessing neurological scores, it was observed that rats subjected to 1 h, 90 min, and 2 h of MCAo + reoxygenation without EVOO treatment exhibited a Modified Garcia score with a 13/18 average. In contrast, treated rats displayed a significantly improved Modified Garcia score, averaging 17/18 [32,33,34,35].

### 2.4. Oxygraphy

**Figure 4:**
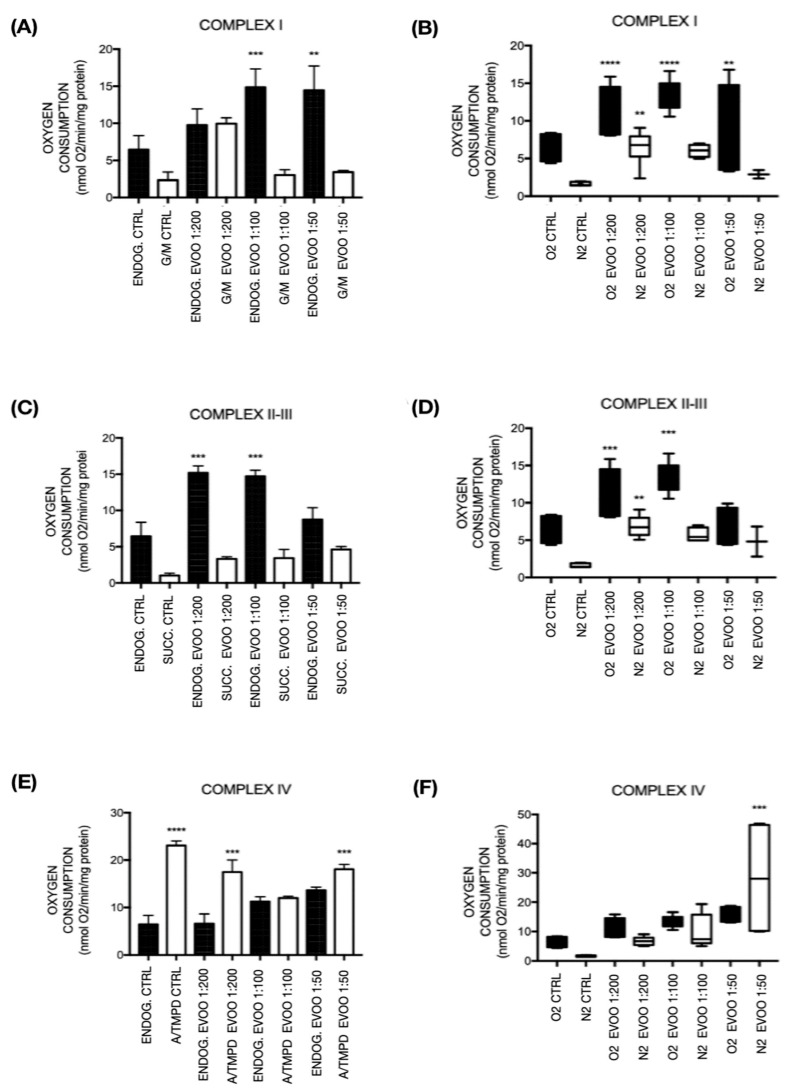
Extra-virgin olive oil (EVOO) treatment in TNC WT and TNC M23 cells. (**A**) TNC wild-type (WT) and (**B**) TNC M23 cells treated with EVOO to assess mitochondrial respiration via nicotinamide adenine dinucleotide (NAD⁺)-dependent substrates; (**C**) TNC WT and (**D**) TNC M23 cells treated with EVOO for mitochondrial respiration by succinate; (**E**) TNC WT and (**F**) TNC M23 cells treated with EVOO for mitochondrial respiration by ascorbate + N, N, N’, N’-tetramethyl-p-phenylenediamine (A/TMPD). Treatments were conducted over 24 h at dilutions of 1:200, 1:100, and 1:50. Statistically significant increases in oxygen consumption are observed, with rates normalized to protein content in a 0.1 mL solution. Statistical analysis was performed using ANOVA (Analysis of Variance) followed by a Newman–Keuls multiple comparison post-test; n = 3. Treatments with statistical significance are indicated with asterisks representing *p*-values: ** *p* < 0.01; *** *p* < 0.001; **** *p* < 0.0001. ENDOG. = endogenous; SUCC. = succinate; G/M = glutamate/malate; A/TMPD = ascorbate/N, N, N’, N’-tetramethyl-p-phenylenediamine.

Under physiological conditions, TNC WT and TNC M23 astrocyte cells treated with EVOO exhibit a noteworthy oxygen consumption elevation compared to untreated cells. Our assessment included an endogenous cellular respiration examination, which denotes respiration without the addition of any substrate, and cellular respiration after the introduction of various substrates. Particularly within complex I and complexes II-III, we observed a significant augmentation in endogenous respiration among treated cells (Figure 4).

#### 2.4.1. Complex I

In the oxygen consumption assessment for Complex I, glutamate/malate (G/M) was employed as the substrate. EVOO-treated TNC WT cells demonstrated a notable elevation in oxygen consumption compared to the untreated control group. Specifically, there was a significant increase in oxygen consumption in cells treated with 1:100 (*p* < 0.001) and 1:50 (*p* < 0.01) EVOO dilutions under endogenous conditions compared to endogenous control. Moreover, a significant increase in oxygen consumption was observed in the 1:200 EVOO-treated dilution group with G/M substrate compared to the G/M control. Interestingly, cells treated with 1:100 and 1:50 EVOO dilutions under endogenous conditions exhibited a reduced oxygen consumption response relative to 1:200 dilution. This trend suggests a dose-dependent effect where higher concentrations (1:100 and 1:50 dilutions) may lead to a reduction or absence of a stimulatory effect on oxygen consumption, which is more pronounced at the 1:200 dilution. This indicates that the EVOO’s efficacy in enhancing oxygen consumption may diminish at higher concentrations (Figure 4A). In TNC M23 cells, an increase in mitochondrial respiration can be observed in cells treated with 1:200 and 1:100 EVOO dilutions compared to the control under ischemic conditions, with respiration similar to the control in oxygen, suggesting protection by EVOO to the cells during hypoxia (Figure 4B).

#### 2.4.2. Complex II-III

Succinate was employed as the substrate to assess oxygen consumption. In TNC WT, the preceding graph illustrates a significant increase in oxygen consumption in EVOO-treated samples compared to the control. Specifically, there is a substantial increase in oxygen consumption in cells treated with 1:200 (*p* < 0.001) and 1:100 (*p* < 0.001) endogenous EVOO compared to the endogenous control, followed by an increase in 1:50 dilution endogenous EVOO-treated cells. Additionally, upon the addition of the substrate succinate, elevated oxygen consumption was evident in all EVOO-treated groups (1:200, 1:100, and 1:50 dilutions) when compared to the succinate control. This progressive rise in oxygen consumption suggests that EVOO treatment enhances mitochondrial activity in a dose-dependent manner, with the 1:200 and 1:100 dilutions showing the most pronounced effects. The increase observed with the 1:50 dilution, although significant, is comparatively lower, indicating that higher concentrations may have a diminishing stimulatory effect on oxygen consumption in Complexes II–III (Figure 4C). In TNC M23 cells, we can also observe an increase in mitochondrial respiration in EVOO-treated cells under ischemic and physiological conditions, especially with 1:200 and 1:100 EVOO dilutions compared with the controls. It can also be seen that cells treated with the 1:200 and 1:100 EVOO dilutions under ischemic conditions exhibited respiration similar to the control in oxygen, suggesting a protective effect by EVOO in cells placed in hypoxia (Figure 4D).

#### 2.4.3. Complex IV

The substrate ascorbate/N, N, N’, N’-tetramethyl-p-phenylenediamine (A/TMPD) was utilized. Notably, TNC WT EVOO-treated cells demonstrated lower oxygen consumption at all dilutions compared to the A/T control. However, there was a significant increase in oxygen consumption in cells treated with 1:100 (*p* < 0.001) and 1:50 (*p* < 0.001) endogenous EVOO dilutions compared to the endogenous control. This suggests that while the EVOO treatment reduces overall oxygen consumption relative to the A/T control, it enhances oxygen consumption in a dose-dependent manner under endogenous conditions (Figure 4E). In TNC M23 cells, an increase in mitochondrial respiration can be observed in EVOO-treated cells, particularly in EVOO 1:100 and 1:50 dilutions under ischemic and physiologic conditions (Figure 4F).

**Figure 5:**
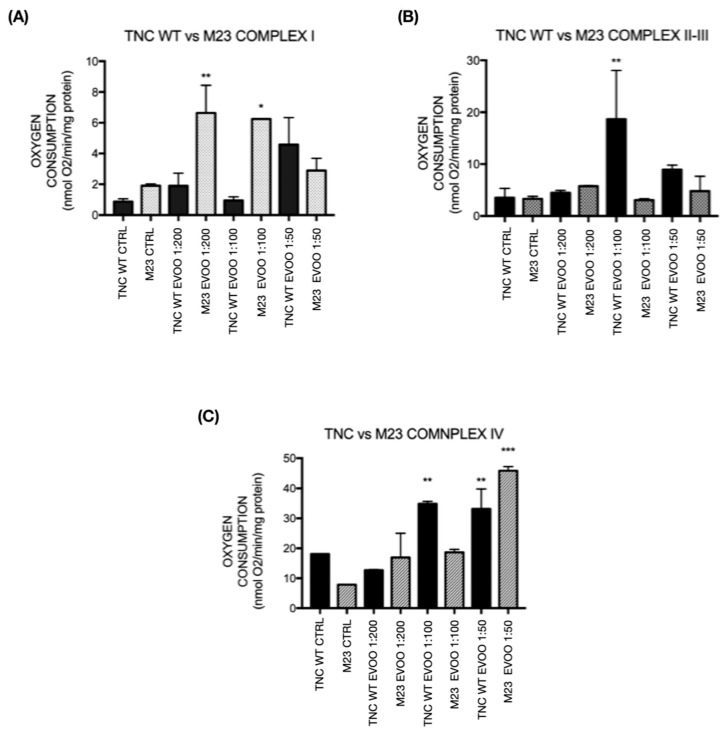
Extra-virgin olive oil (EVOO) treatment in comparison between TNC WT and TNC M23 cells. (**A**) EVOO effect on mitochondrial respiration via nicotinamide adenine dinucleotide (NAD⁺)-dependent substrates. (**B**) Succinate effect on mitochondrial respiration. (**C**) Ascorbate + N, N, N’, N’-tetramethyl-p-phenylenediamine effect on mitochondrial respiration (TMPD). Treatments were conducted over 24 h at dilutions of 1:200, 1:100, and 1:50. A statistically significant increase in oxygen consumption is observed, with rates normalized to protein content in a 0.1 mL solution. EVOO = olive oil. Statistical analysis included ANOVA (Analysis of Variance) followed by a Newman–Keuls multiple comparison post-test; n = 3. Statistically significant treatments are marked with asterisks indicating *p*-values: * *p* < 0.05; ** *p* < 0.01; *** *p* < 0.001.

Our findings revealed a significant increase in oxygen consumption in TNC M23 cells compared to TNC WT cells at complex I (Figure 5A). 

In complexes II and III, the oxygen consumption was comparable between the two cell lines, except for TNC WT cells treated with 1:100 EVOO dilution, which showed a significant increase (Figure 5B). For complex IV, an increase in respiration was observed in TNC WT cells treated with 1:100 EVOO compared to TNC M23 cells. However, in the case of the 1:50 EVOO dilution, TNC M23 cells exhibited a more pronounced increase in oxygen consumption than TNC WT cells (Figure 5C).

### 2.5. ROS Measurement

**Figure 6:**
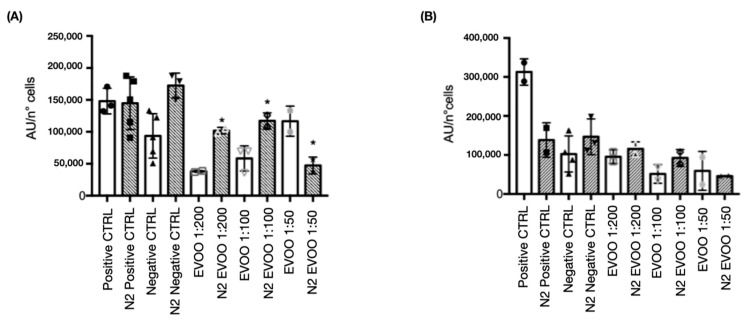
Extra-virgin olive oil (EVOO) treatment effects on ROS production under physiological and hypoxic conditions. (**A**) TNC wild-type (WT) and (**B**) TNC M23 cells treated with EVOO for 24 h at dilutions of 1:200, 1:100, and 1:50. During this period, cells were subjected to 5 h of ischemic conditions followed by 19 h of reoxygenation. A statistically significant reduction in reactive oxygen species (ROS) production was observed in EVOO-treated cells, with ROS levels normalized to cell count in each 96-well plate. Statistical analysis included ANOVA (Analysis of Variance) with a Newman–Keuls multiple comparison post-test; n = 3. Significant treatments are marked with asterisks, with *p*-value ranges as follows: * *p* < 0.05.

In the ROS measurement, EVOO-treated TNC WT cells produced more ROS during hypoxia compared to non-EVOO-treated cells. This reduction in ROS production was particularly significant in cells treated with 1:200 (*p* < 0.05) and 1:100 (*p* < 0.05) EVOO dilutions. These findings indicate that EVOO treatment leads to elevated ROS production during hypoxia; it also effectively mitigates ROS levels compared to untreated hypoxic cells, demonstrating a protective effect of EVOO under stress conditions (Figure 6A). In TNC M23 cells, a slight decrease in ROS production was observed in EVOO-treated cells compared to control and TNC WT cells (Figure 6B).

### 2.6. Viability Assay

**Figure 7:**
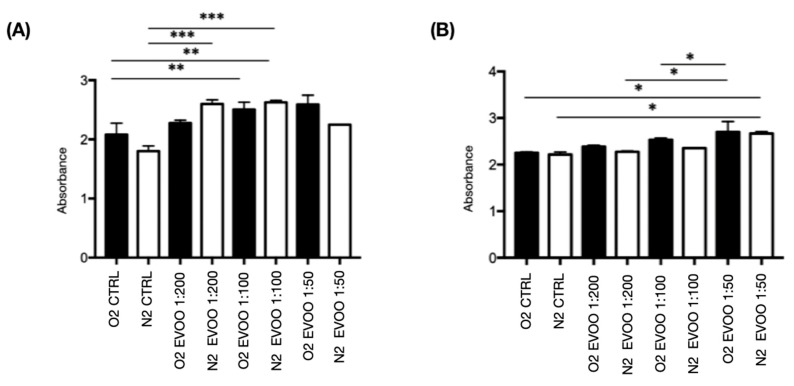
Extra-virgin olive oil (EVOO) effects on vitality cells under physiological and hypoxic conditions. (**A**) TNC WT and (**B**) TNC M23 cells with EVOO treatment were conducted for 24 h at different dilutions (1:200-1:100-1:50) within 24 h and placed for five h under ischemic conditions + reoxygenation in the remaining 19 h. A statistically significant increase in cell vitality in EVOO-treated cells can be observed. The vitality test was normalized to the cell number in 96 wells. A multiple comparison Newman–Keuls post-test was used after performing an ANOVA (Analysis of Variance); n = 4. The treatments that resulted in statistical significance after the statistical analysis were marked with asterisks, which in the legend are explained as the *p*-value ranges = * *p* < 0.05; ** *p* < 0.01; *** *p* < 0.001.

In the viability assay conducted with TNC WT cells under physiological and ischemic conditions (5 h of hypoxia followed by 24 h of reoxygenation), a minor decrease in cell count was observed in the hypoxia control compared to the oxygen control. Additionally, EVOO-treated cells showed comparable absorbance levels under oxygenated and hypoxic conditions, indicating that the EVOO protects against cell death induced by hypoxia. This suggests a beneficial effect of EVOO treatment in preserving cell viability during hypoxic stress (Figure 7A).

As for TNC M23 cells, the behavior was similar to TNC WTs, with a difference in that the hypoxia control showed a slight difference in absorbance to the oxygen control and that, in this case, the EVOO acted on the cells under physiological conditions by stimulating their reproduction compared with the oxygen control (Figure 7B).

### 2.7. XF Real-Time ATP Rate Assay

The XF Real-Time ATP Rate Assay allowed us to quantify mitochondrial ATP production by injection of Oligomycin, an inhibitor of mitochondrial ATP synthesis, which induces a reduction in OCR. These factors were combined with ECAR data to calculate the total PER. Complete mitochondrial respiration inhibition with Rotenone plus Antimycin A allows for mitochondria-associated acidification and, when combined with PER data, allows for the glycoATP production rate calculation.

**Figure 8:**
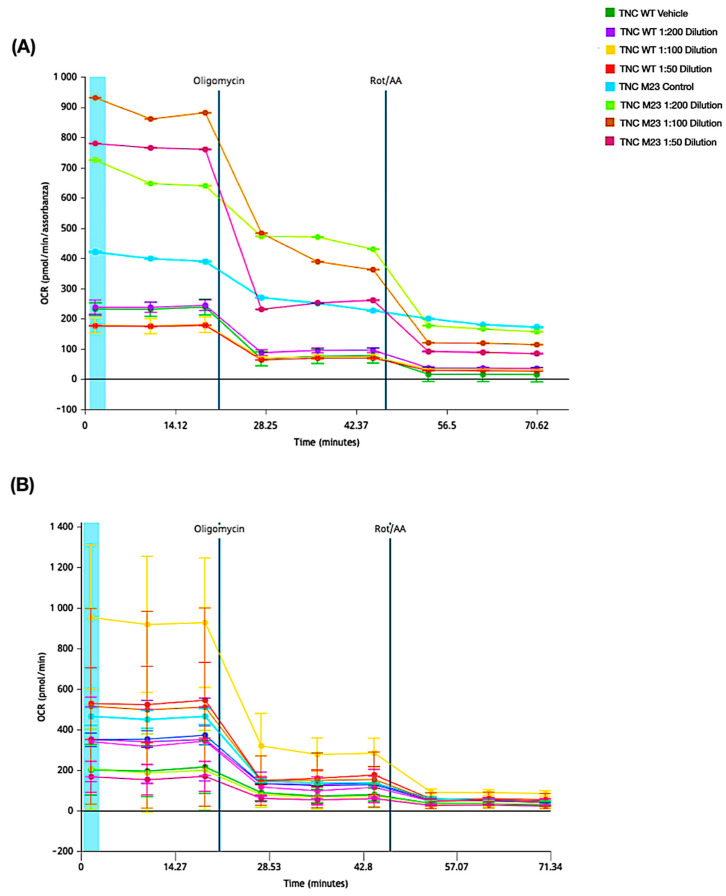
Extra virgin olive oil (EVOO) on ATP production. The kinetic graph shows the basal oxygen consumption rate (OCR) under (**A**) physiological and (**B**) hypoxic conditions. Injection of Oligomycin inhibits mitochondrial ATP synthesis, leading to a decrease in OCR and enabling quantification of the mitochondrial ATP (mitoATP) production rate. Complete mitochondrial respiration inhibition by Rotenone plus Antimycin A accounts for mitochondrial-associated acidification. Combined with proton efflux rate (PER) data, this allows for the calculation of the glycolytic ATP (glycoATP) production rate.

It can be seen that cells under ischemic conditions, TNC WT produce more ATP when treated with a 1:100 EVOO dilution compared with control and other treatments and also show reduced EVOO acidification. In TNC M23, a progressive reduction in ATP production in treated cells was observed compared with the control, presenting a markedly different behavior from TNC WT (Figure 8A).

Under physiological conditions, TNC WT cells produce less ATP than TNC M23s, with similar production in cells treated with 1:200 EVOO dilution and the control, and a slight decrease in cells treated with 1:100 and 1:50 EVOO dilutions. For TNC M23s, a markedly higher ATP production in EVOO-treated cells is observed compared with the control, but with higher lactate production in the 1:200 EVOO dilution compared with the control and the other treatments, which show markedly less acidification instead (Figure 8B).

## 3. Discussion

Cerebral ischemia represents a pathological state characterized by blood and oxygen deprivation at the neuronal tissue level. This condition leads to the brain cells’ impairment or destruction and instigates various deleterious consequences. The responses to injury and disease within the CNS entail multiple coordinated neural and non-neural cell type interactions over time. This interaction is crucial for maintaining homeostasis, safeguarding viable cells, clearing debris, and preserving overall function [2,4,36]. Among these responses, astrocytes emerge as indispensable responders to diverse forms of CNS insults, manifesting dynamic changes collectively recognized as reactive astrogliosis [37].

Various studies have documented the impact of ischemia on astrocytic metabolic pathways. During ischemic events, astrocytes undergo significant metabolic adaptations to support neuronal survival and maintain brain homeostasis [5,12]. One of the key adaptations involves glycogen metabolism, as astrocytes serve as the primary glycogen reservoir in the brain [14]. Under ischemic stress, glycogen is rapidly mobilized through glycogenolysis to provide an alternative energy source in the form of lactate, which is shuttled to neurons via monocarboxylate transporters [14]. This lactate supply helps sustain neuronal ATP production when glucose availability is compromised. Additionally, ischemia induces an upregulation of glycogen synthesis enzymes as part of a compensatory mechanism to replenish energy stores once perfusion is restored [10]. However, excessive glycogen accumulation can also be detrimental, as its breakdown leads to lactate acidosis, exacerbating cellular damage. Interestingly, bioactive compounds present in EVOO, such as polyphenols and oleocanthal, have been shown to modulate glucose and glycogen metabolism, reducing oxidative stress and inflammation, thereby enhancing astrocytic resilience during ischemia [27].

In addition to glycogen metabolism, lipid metabolism in astrocytes is also significantly altered during ischemia, influencing brain homeostasis. Under hypoxic conditions, astrocytes increase lipolysis and fatty acid oxidation to generate ATP, compensating for the diminished glucose supply [14]. Concurrently, ischemia disrupts lipid trafficking and peroxidation pathways, leading to an accumulation of ROS and lipid peroxidation byproducts, which can exacerbate oxidative stress and inflammatory responses. Astrocytes also modulate phospholipid composition to preserve membrane integrity, ensuring the proper function of ion channels and transporters critical for neuronal support [13]. Notably, EVOO-derived polyphenols have demonstrated neuroprotective effects by mitigating lipid peroxidation and reducing ROS-mediated astrocytic dysfunction [30]. Furthermore, EVOO components, such as oleic acid and hydroxytyrosol, support astrocytic lipid metabolism by enhancing mitochondrial function and promoting anti-inflammatory pathways [31]. These metabolic shifts highlight the crucial role of astrocytes in adapting to ischemic stress while also suggesting that dietary interventions, such as EVOO consumption, may serve as a potential strategy to enhance astrocytic resilience and neuroprotection during ischemic events.

Following focal cerebral ischemia in rats, a time-dependent reduction in glucose oxidation was observed, concomitant with the heightened oxidative metabolism of neuronal Gamma-aminobutyric acid (GABA) and glutamate. Furthermore, there was an increased transfer of glutamine to neurons [38]. These findings substantiate the key role of astrocytic glutamate uptake in curtailing neuronal death induced by excitotoxic glutamate effects [39,40,41], achieved through its conversion to glutamine. While robust evidence is currently lacking, it is posited that astrocytes may furnish neurons with antioxidant substrates, including glutathione. Additionally, the heightened lactate release by astrocytes during ischemia has been suggested as a potential neuronal fuel source during the recovery phase [6,13].

Mitochondria are crucial in energy production and ROS generation, particularly in the ischemic injury context. Our study observed a notable increase in oxygen consumption within Complex I of EVOO-treated TNC WT cells, particularly evident at 1:100 and 1:50 endogenous EVOO dilutions, with a significant rise also seen at the 1:200 dilution compared to the respective controls, suggesting potential diminishing effects at higher concentrations. In TNC M23 cells, an increase in mitochondrial respiration can be observed in cells treated with 1:200 and 1:100 EVOO dilutions compared to the control under ischemic conditions, with respiration similar to control in oxygen, suggesting protection of the cells by EVOO during hypoxia.

Mitochondrial Complex I plays a key role in brain ischemia pathophysiology, as highlighted by Beal et al. [42] who underscored its involvement in neurodegenerative diseases and ischemic damage processes. Complex I impairment reduced ATP production and heightened ROS generation, exacerbating neuronal injury. This dysfunction elevated oxidative stress, damaging lipids, proteins, and DNA in ischemic brain tissue [43]. Moreover, Di Lisa and Bernardi [44] emphasized Complex I’s critical role in regulating mitochondrial function during ischemia, where its dysfunction compromises calcium homeostasis and increased mitochondrial permeability, leading to cell death pathways. Sims and Anderson [45] and Huang and Carlson [46] further explored how Complex I dysfunction correlates with increased oxidative damage and poor outcomes in ischemic stroke models, underscoring its potential as a therapeutic target to mitigate ischemic injury and enhance neuronal survival.

In our investigation of mitochondrial Complexes II and III, in both cell lines, we observed a progressive increase in oxygen consumption across various endogenous EVOO dilutions (1:200, 1:100, and 1:50) compared to controls, particularly notable upon succinate supplementation. This finding suggests that the EVOO treatments may influence mitochondrial function by altering electron transport dynamics and subsequent oxygen utilization. Gellerich et al. [17] underscored the role of Complex II dysfunction in ischemic conditions, where impaired succinate dehydrogenase activity leads to succinate accumulation, exacerbating ROS production and oxidative stress during reperfusion phases,

Complex III dysfunction under ischemic conditions disrupts electron flow within the electron transport chain, impairing ATP production and enhancing superoxide generation, thus contributing to oxidative damage and neuronal injury [18]. Dietrich et al. [47] explored protective strategies targeting Complexes II and III, suggesting that interventions preserving their function could mitigate mitochondrial damage, reduce ROS levels, and potentially enhance neuronal survival post-ischemia. Furthermore, Tretter et al. [48] highlighted Complex III’s vulnerability to ischemia–reperfusion injury, emphasizing the importance of targeted therapeutic approaches to safeguard mitochondrial function and improve outcomes in ischemic brain injury.

In our investigation, in TNC WT cells, we observed relatively lower oxygen consumption in EVOO-treated cells across all dilutions when focusing specifically on Complex IV, compared to the A/T control group. However, there was a noticeable increase in oxygen consumption at the 1:100 and 1:50 endogenous EVOO dilutions compared to the endogenous control, indicating a potential modulatory EVOO treatment effect on Complex IV function. In TNC M23 cells, an increase in mitochondrial respiration could be observed in EVOO-treated cells, particularly with EVOO 1:100 and 1:50 dilutions under ischemic and physiological conditions. Li et al. explored ischemia’s impact on Complex IV activity across different brain regions, revealing varying degrees of damage to cytochrome C oxidase activity among regions. This regional variability underscores the importance of understanding localized responses to ischemic injury and tailoring therapeutic strategies accordingly [49].

Rasmussen et al. [50] investigated the ischemic protective effects of preconditioning on Complex IV function in brain tissue, demonstrating that brief periods of ischemia could precondition cells and preserve Complex IV activity. This preservation was associated with improved cellular resilience and better outcomes following ischemic brain injury. Additionally, Hüttemann et al. [51] delved into the molecular mechanisms underlying Complex IV dysfunction during ischemia, highlighting post-translational modifications of cytochrome c oxidase subunits as contributors to impaired enzyme function. Their findings suggested potential targets for therapeutic interventions aimed at preserving Complex IV activity and mitigating ischemic damage.

Furthermore, Zhu et al. [52] explored mitochondrial biogenesis as a strategy to enhance the recovery of Complex IV function post-ischemia. Their studies indicated that promoting mitochondrial biogenesis could effectively restore Complex IV activity and improve mitochondrial respiration in ischemic brain tissue, offering promising avenues for reducing long-term damage and enhancing recovery after ischemic events.

Chen et al. [53] investigated ATP synthase in ischemic brain tissue and found a significant reduction in its activity during ischemia, correlating with decreased ATP levels and cellular energy failure under ischemic conditions. Kushnareva et al. [20] explored the impact of ischemia on ATP synthase subunit composition and assembly, revealing disruptions that impair enzyme function and decrease ATP production in ischemic brain regions. Duchen et al. [54] emphasized ATP synthase’s role in maintaining mitochondrial membrane potential and preventing the opening of the mitochondrial permeability transition pore (mPTP) during ischemia, which is crucial for preserving mitochondrial integrity and averting cell death pathways triggered by ischemic insults. Abe et al. investigated therapeutic strategies targeting ATP synthase activity in ischemic stroke, proposing that enhancing ATP synthase function or stabilizing its structure could shield neurons from ischemic injury and enhance recovery post-stroke [55].

Furthermore, Bélanger et al. [56] explored the link between ATP synthase dysfunction and mitochondrial oxidative stress in ischemic brain injury, demonstrating that impaired ATP synthase activity contributes to heightened ROS production during ischemia, exacerbating neuronal damage.

Mitochondria emerge as a significant target in H/I injury, and mitochondrial homeostasis disturbance and cellular energetics exacerbate the H/I insult outcomes, particularly in elderly individuals. In response to acute injury conditions, cellular machinery promptly adapts through modulating posttranslational modifications. Oxygen and nutrient deficiencies resulted in reduced reliance on mitochondria for energy, promoting glycolysis [57].

The oxidative stress occurrence ensues when the system fails to maintain equilibrium between the production and utilization of oxidant molecules. Excessive ROS generation during oxidative stress can induce cellular injury by causing damage to proteins, DNA, and lipids, as evidenced in I/R models [58,59].

In ROS measurement, EVOO-treated TNC WT cells produced more ROS during hypoxia compared to non-hypoxia-treated cells. This reduction in ROS production was particularly significant in cells treated with 1:200 and 1:100 EVOO dilutions. In TNC M23 cells, a slight decrease in ROS production was observed in EVOO-treated cells compared to control and TNC WT cells.

ROS are generated both within and outside the mitochondria. Extra-mitochondrial sources of ROS encompass lipoxygenases, peroxisomes, and NADPH oxidases [60]. However, the primary ROS production site is within the mitochondria, with complexes I and III being the predominant sites. Despite the common attribution of mitochondrial ROS to aging, there is evidence to the contrary [15,16,17,18]. Superoxide is an inevitable by-product of mitochondrial electron transport at complexes I–III [18]. The endogenous antioxidants glutathione peroxidase, catalase, and superoxide dismutase play crucial roles in scavenging free radicals. In these scavengers’ absence, ROS induces deleterious effects on cellular functions. Consequently, mitochondria play an important role in H/I injury and contribute to the age-dependent decline in metabolic processes and organ function [16].

N-3 PUFAs serve as neurotrophic factors renowned for their beneficial roles in neurodevelopment. Prior research has demonstrated that elevated levels of n-3 PUFAs protect the adult and neonatal brain against ischemic brain damage through multiple mechanisms. These mechanisms include inflammatory response suppression, oxidative stress alleviation, neurovascular unit reconstruction enhancement, and oligodendrogenesis promotion [60,61,62,63,64,65,66,67,68]. The action of n-3 PUFAs permits the stabilization of the myocyte membrane, attributed to the partial inhibition of voltage-dependent sodium and calcium channels, along with the inhibition of potassium channels [60]. These observations sparked initial interest in potential antiarrhythmic therapy involving these fatty acids [62]. A similar effect was identified in a treatment with various anticonvulsant drugs (valproic acid, carbamazepine, lamotrigine, phenytoin), which also induced the partial inhibition of voltage-dependent sodium and calcium ion channels. Consequently, n-3 PUFAs have been associated with a potential anticonvulsant action [65].

In the brain, DHA stands as the most important n-3 PUFA, serving as a key structural component of neuronal membranes and playing a vital role in regulating neuronal functions, including interactions with ion channels, modulating neurotransmitter release, and other functions. and essential processes [60,68]. DHA has demonstrated protective effects against blood–brain barrier (BBB) disruption after focal cerebral ischemia in adult rats [47]. Although n-3 PUFAs have been shown to provide neuroprotection in neonatal H/I injury, it remains unknown whether n-3 PUFAs can preserve BBB integrity in the event of injury, as the precise mechanisms underlying this protection are not yet well understood [69].

Certain specific components, such as DHA, are of extreme nutritional importance in any diet type. The Mediterranean diet, which features regular fish consumption, is recognized for its health benefits and is distinguished by bioactive compounds such as monounsaturated fatty acids (MUFAs), PUFAs, and polyphenols. It has already been proven that this diet component modulates responses to inflammatory or oxidative mediators [70].

Notably, moderate fish and seafood consumption is well-known to impact cerebrovascular mortality [7], and it is associated with a reduced incidence of subclinical brain infarcts [9]. Fish, a significant omega-3 fatty acids source, particularly eicosapentaenoic acid (EPA) and DHA, is believed to confer cardiovascular benefits and is linked to a lower risk of death from cardiovascular disease [68]. EVOO, rich in oleic acid, a non-essential monounsaturated fatty acid, is associated with a lower ischemic stroke risk, suggesting potential neuroprotective effects [32]. EVOO has been shown to protect against neuronal cell death in rodent cerebral ischemia models, ameliorating brain injury [28].

Several studies support n-3 PUFA’s beneficial effects [71,72]. Our findings align with those of Ek et al. [69], who described in their research that the pathology of H/I brain injury involves BBB disruption. This disruption contributes to cerebral edema and secondary neuronal injury. As a measure of edema, hemispheric water content was examined 48 h after H/I injury. Animals on a normal diet (N3L) and supplemented with n-3 PUFA (N3H) exhibited an increased brain water content and indicated significant brain edema following H/I compared to sham controls. However, n-3 PUFA treatment notably reduced water content in the ipsilateral brain tissue.

In a related context, Zhang et al. [70,73] emphasized tight junction proteins in maintaining BBB integrity. After H/I injury, tight junction protein expression, such as occludin, ZO-1, claudin-5, and cadherin-10, was significantly decreased. However, n-3 PUFA treatment preserved the expression of these proteins. As anticipated, n-3 PUFA dietary supplementation markedly maintained their in situ expression. Furthermore, in another study, Zhang et al. [74] demonstrated that the ischemic area size in mice fed with n-3 PUFAs was lower than that in groups not fed with n-3 PUFAs, indicating a neuroprotective effect of the diet enriched in DHA, EPA, and MUFAs from olive EVOO.

Despite several strengths, our study has limitations that must be considered. Our study did not perform bioavailability assessment assays, and the dilutions were decided based on the literature in animal model studies. This study utilized only three EVOO dilutions, suggesting that future research with a more comprehensive analysis is warranted. Moreover, while the research attributes EVOO’s protective effects to n-3 PUFA acids, it is crucial to acknowledge the presence of other bioactive components in EVOO. These components include polyphenols, tocopherols, sterols, and several minor components that may also contribute significantly to the antioxidant properties and overall health benefits associated with EVOO consumption. Synergistic interactions need to be investigated in future studies to better understand these aspects.

## 4. Materials and Methods

### 4.1. EVOO Chemical Structure

The EVOO used was a natural extraction type made in Puglia, Italy (“Tesserae” EVOO, Frantoio Famiglia Di Palma” Canosa di Puglia, Italy). Structure and composition information can be found in Supplementary Material Table S1.

#### 4.1.1. Chemicals and Standards

LC-MS grade MeOH, formic acid, and Quinaldic acid, which was used as an internal standard, were purchased from Sigma Aldrich (Darmstadt, Germany). The Phenolic Acids and Alcohols Standard Mixture V2 and the Flavonoids Standard Mixture V2 were purchased from MetaSci library (Toronto, ON, Canada, https://www.metasci.ca/) and were used for peak identification, MRM method development, and making calibration curves for quantification.

#### 4.1.2. LC-MS/MS Analysis of Phenolic Acids and Flavonoids

The LC-MS/MS system consisted of a UHPLC (Nexera Series LC-40, Shimadzu, Kyoto, Japan) coupled to a triple quadrupole/linear ion trap tandem mass spectrometer (QTRAP 4500, AB Sciex, Framingham, MA, USA) equipped with a Turbo V ion source. Instrument control, data acquisition, and processing were performed using the associated Analyst 1.6 and Multiquant 3.0 software (AB Sciex, Framingham, MA, USA). The LC separation was performed using a Gemini C6-Phenyl 110Å column (50 × 2.0 mm, particle size 5 µm) from Phenomenex (Torrance, CA, USA). Elution was performed at a flow rate of 700 µL/min with water containing 0.1% (*v*/*v*) formic acid as eluent A and MeOH (Merck, Darmstadt, Germany) containing 0.1% (*v*/*v*) as eluent B. Two slightly different gradient chromatographic methods were developed for phenolic acids and flavonoids. For the former metabolite class after a column equilibration time of 3 min: 0–5 min, from 2 to 30% of phase B; 5–7 min, 30–100% of phase B; 7–9 min holding the phase B concentration; 9–10 min back to initial condition of 2% of phase B. For flavonoids, after a column equilibration time of 2 min, we employed the following: 0–5 min, from 2 to 30% of phase B; 5–7 min, 30–100% of phase B; 7–9 min holding the phase B concentration; 9–12 min back to initial condition of 2% of phase B. The column oven was set to 40 °C, and 2 mL of the samples was injected. The Q1 resolution was adjusted to 0.7 ± 0.1 amu fwhm for MRM, referred to as the unit resolution. Q3 was also set to the unit resolution in MRM mode. Two scheduled MRM (sMRM) methods were developed, one for phenolic acids containing 43 metabolites and one for flavonoids containing 38 metabolites for 81 polyphenols. MS analysis was carried out in negative and positive ionization modes using an ion spray voltage of ±4500 V. The nebulizer and the curtain gas flows were set at 35 psi using nitrogen. The Turbo V ion source was operated at 350 °C for phenolic acids and 450 °C for flavonoids. Ion Source Gas 1 and 2 were both set at 40 psi. Suitable MRM transitions were selected for the targeted metabolites and Quinaldic acid, which were used as the internal standard (IS). The compound-dependent parameters for metabolites and IS were optimized using the manual optimization protocol in tuning mode. The Q1 mass, the Q3 transition, the best parameters, and the retention time are reported in Table 3. Quinaldic acid was spiked to each sample solution at a final 0.5 µg/mL concentration. Equal volumes of each extract were combined to create three pooled quality control (QC) samples. The pooled QC samples and the Quinaldic acid area values were utilized to monitor the quality and robustness of the analysis. QC samples were injected at three points during the batch: at the beginning, middle, and end of the run.

#### 4.1.3. Method Validation

Method validation was conducted by analyzing calibration curves, limits of detection (LOD), and limits of quantification (LOQ). Independent calibration curves were constructed for phenolic acids and flavonoids using the serial dilution method. The calibration curves were generated by plotting the metabolite area of the selected sMRM transition against the concentration of the metabolite solution. The linearity range of each metabolite was assessed using eight calibration points covering a concentration range of 0.003 to 10.0 µg/mL. Each point was analyzed in triplicate. The linearity range, curve equations, LOD, and LOQ are summarized in Table 4. LOD and LOQ were calculated as 3 times and 10 times the standard deviation of the blank.

#### 4.1.4. Fatty Acid Analysis

The derivatization and quantification of fatty acids were performed at “EOS LAB s.r.l”, Foggia, Italy. Briefly, fatty acids were dissolved in heptane (1 mL) and transesterified by adding 0.15 mL of KOH in methanol (10%) and stirring for 5 min. Then, the phases were allowed to separate, and the heptane phase was taken for analysis by GC-FID. The fatty acid profile analysis was carried out in a PerkinElmer Mod Clarus 500 Gas Chromatograph (Waltham, MA, USA), equipped with a 60-m BPX7 capillary column, 0.25 mm internal diameter, and 0.25-micron phase thickness. Further, 1 µL of sample was injected under the following chromatographic conditions: injector temperature, 235 °C; column oven, 170 °C (10 min); ramp of 5 °C per minute up to 235 °C for 3 min; temperature detector FID 250 °C. The GC was used in the constant pressure mode at 25 psi of hydrogen. Identification of the methyl esters of fatty acids was performed by comparing the retention times with a reference mix standard containing 55 fatty acids and taking the relative mean absolute error at 1.5%. The percentage of individual fatty acids was calculated in relation to the total area of the chromatogram (fatty acid profile), working with the area of each compound (Figure 2).

### 4.2. In Vivo Model

All in vivo experiments were conducted at Loma Linda University’s Center for Neuroscience Research. Supplementary Material S2 thoroughly describes the methodology. Institutional Animal Care and Use Committee (IACUC) approval was obtained from Loma Linda University (code: 20-003) following the National Institute of Health guidelines for laboratory animal care. Sixty male Sprague Dawley (SD) breed rats (260–280 g body weight) were used in controlled conditions with proper temperature and humidity conditions, regular light/dark cycles, and unrestricted access to water and food.

The rats were randomly assigned to ten groups undergoing distinct experimental conditions involving middle cerebral artery occlusion (MCAo), recanalization, and EVOO consumption. For each group, four rats were used for mitochondrial isolation, and two were used for TTC staining. The groups’ administration was as follows: 1. aham; 2. MCAo 1 h + recanalization 24 h; 3. MCAo 90 min + recanalization 24 h; 4. MCAo 2 h + recanalization 24 h; 5. MCAo 1 h + recanalization 24 h with EVOO at 0.5 mL/kg/day; 6. MCAo 1 h + recanalization 24 h with EVOO at 1 mL/kg/day; 7. MCAo 90 min + recanalization 24 h with EVOO at 0.5 mL/kg/day; 8. MCAo 90 min + recanalization 24 h with EVOO at 1 mL/kg/day; 9. MCAo 2 h + recanalization 24 h with EVOO at 0.5 mL/kg/day; 10. MCAo 2 h + recanalization 24 h with EVOO at 1 mL/kg/day. The decision on the EVOO amount used in vivo was made according to the “ADME” principle (Absorption, Distribution, Metabolism, and Excretion) and according to descriptions from Beckers et al., Galmés et al., and Nikou et al. studies that elucidate the bioavailability and systemic exposure of key phenolic compounds after oral administration [29,30,31].

Oral gavage, a method for administering a specific agent volume orally, was employed using each described dosage before surgery. Neurological impairments were assessed by blindly evaluating the Modified Garcia score and beam walking test results [32].

The beam walking test gauged the rat’s coordination and ability to traverse a narrow wooden beam for 60 s [33]. The infarct area and the ipsilateral hemisphere of each brain slice were quantified using ImageJ https://ij.imjoy.io/ (Laboratory for Optical and Computational Instrumentation, LOCI, University of Wisconsin, USA). The results were in the form of a corrected infarct volume ratio to full contralateral hemispheric volume [34].

The rats were euthanized, and their brains were promptly bifurcated into two hemispheres: the left ischemia side and the right control side. The mitochondria were isolated and analyzed.

### 4.3. In Vitro Model

#### 4.3.1. Cell Culture

The astrocyte cell lines used were wild type (WT) Telencephalic Neural Cells (TNC) and TNC M23, isoforms of the water channel aquaporin-4 (AQP4), extracted from the rat diencephalon (kindly provided by Professor Antonio Frigeri, University of Bari, Italy). TNC M23 is an astrocyte cell transfected with the target “p” plasmid that introduced a DNA M23 to allow for AQP4 expression, which is normally absent in TNC WT. These cells were cultured under normoxic and hypoxic conditions. AQP4 expression is vital in ischemia/reperfusion (I/R) studies because it plays a crucial role in maintaining water homeostasis in the brain, particularly in astrocytes. Normoxic conditions were maintained in a CO_2_/O_2_ incubator set to 37 °C, with a humidified atmosphere comprising 5% CO_2_, 93% N2, and 2% O_2_. The initial treatment involved administering EVOO dissolved in dimethyl sulfoxide (DMSO) (Sigma-Aldrich, Saint Louis, MO, USA) at varying dilutions: 1:200, 1:100, and 1:50. The EVOO-DMSO solution was prepared for each experiment to ensure consistency and efficacy.

The dilutions were designed based on previous tests considering a minimum dilution (1:50) that was a total EVOO dilution in DMSO. For other dilutions to be analyzed from this point onwards, double the dilution (1:100) and twice the double dilution (1:200) were considered.

Cells were plated in 60 cm² disposable culture dishes and supplemented with the appropriate growth medium. Following plating, the cells were treated with the prepared EVOO dilutions for 24 h under normoxic conditions. For hypoxia treatment, the cells were exposed to EVOO-DMSO solution for 5 h in an incubator with a reduced oxygen concentration of 2%. This setup aimed to mimic hypoxic conditions. After this initial hypoxic exposure, the cells were returned to normoxic conditions for a reoxygenation period of 19 h to assess their response to reperfusion. The experiments were conducted in triplicate to ensure the results’ reliability and reproducibility. This repetition allowed for anomaly identification and ensured that the data collected were robust and representative of typical cell responses under the given conditions.

DMSO is commonly used in cell culture studies for its ability to dissolve both polar and non-polar compounds, making it a versatile solvent. However, its concentration must be carefully controlled due to its biological activity, including potential anti-inflammatory properties. In research studies, DMSO is typically used at concentrations ranging from 0.1% to 1% (*v*/*v*) to avoid cytotoxic effects while maintaining its solvent capabilities. For instance, 0.1% DMSO was used in our experiments.

#### 4.3.2. Oxygraphy—Oxygen Consumption

After 24 h of incubation under normoxic and hypoxic conditions, cells were prepared for oxygraphy analysis. This preparation involved several steps to ensure the cells were in optimal condition for the procedure. First, cells were enzymatically detached from the culture dishes using trypsin (Sigma-Aldrich, Saint Louis, MO, USA) to ensure complete detachment and single-cell suspension formation. The cell suspension was then transferred into 15 mL centrifuge tubes and centrifuged at 300× *g* for 5 min to form cell pellets. Following centrifugation, the supernatant, including the EVOO medium, was carefully aspirated, leaving behind cell pellets.

For the oxygraphy analysis, the Hansatech model Clark oxygen electrode was used to measure cellular oxygen consumption with high precision. The electrode setup involved placing the Clark oxygen electrode within a specialized electrochemical cell featuring a platinum cathode and a silver anode immersed in an electrolyte solution to create the electrochemical environment. The measurement principle behind this polarographic technique involves applying a voltage between the two electrodes, inducing a current proportional to the amount of oxygen present, thereby enabling the measurement of oxygen consumption. The platinum cathode facilitates the reduction of oxygen, generating a measurable current.

The prepared cell pellets were introduced into the electrochemical cell containing the Clark oxygen electrode. As the cells consumed oxygen, the decrease in oxygen concentration was monitored in real-time by measuring the current generated. This current was quantitatively analyzed to assess the mitochondrial respiratory fluxes, providing insights into the cellular oxygen consumption rates under different experimental conditions.

The results from the oxygraphy analysis were meticulously recorded and analyzed to determine the oxygen consumption rates of cells exposed to normoxic and hypoxic conditions, with and without EVOO treatment. The data provided critical insights into the treated cells’ metabolic activity and respiratory efficiency.

#### 4.3.3. ROS Measurement

For the ROS measurement, the experimental setup began with seeding cells into 96-well plates at an appropriate density to ensure sufficient growth for the experiment. The following groups were included in triplicate: a positive control group treated with 100 μM hydrogen peroxide (H₂O₂) (Sigma-Aldrich, Saint Louis, MO, USA) for 30 min to induce oxidative stress; a negative control group treated with DMSO; blank wells without cells to account for background fluorescence; and treatment groups with cells treated with EVOO at various dilutions (1:200, 1:100, 1:50), with and without Dichlorofluorescein (DCFH) (Sigma-Aldrich, Saint Louis, MO, USA). The 24-h EVOO treatments were conducted under physiological and ischemic conditions (5 h of hypoxia followed by reoxygenation for 19 h).

On the day of the experiment, the entire procedure was carried out in darkness to prevent the photobleaching of fluorescent dyes. The treated Dulbecco’s Modified Eagle’s Medium (DMEM) (Agilent, Santa Clara, CA, USA) was carefully aspirated from the wells, and cells were gently rinsed with phosphate-buffered saline (PBS) (Sigma-Aldrich, Saint Louis, MO, USA) to remove any residual treatment media. Cells were then incubated with 10 μM Dichlorofluorescein Diacetate (DCFH-DA) (Sigma-Aldrich, Saint Louis, MO, USA) in a fetal bovine serum (FBS)-free medium (Sigma-Aldrich, Saint Louis, MO, USA) for 20 min at 37 °C. DCFH-DA is a non-fluorescent compound that diffuses into cells and is hydrolyzed by intracellular esterases to DCFH. DCFH is oxidized by ROS action to form a fluorescent compound named 2′,7′-DCFH.

After incubation with DCFH-DA, the medium was carefully aspirated, and the wells were washed with PBS to remove any excess dye. Fluorescence intensity was measured using a Victor multi-well plate reader, equipped to measure fluorescence at the specific excitation (485 nm) and emission (535 nm) wavelengths for DCFH.

For data normalization, the cells were fixed and stained with Crystal Violet (Sigma-Aldrich, Saint Louis, MO, USA) following fluorescence measurement to normalize the ROS data to the cell number. The Crystal Violet stain binds to cellular DNA, providing a cell density measure. After staining, the plates were washed, and the dye was solubilized with acetic acid. Absorbance was measured at 590 nm to quantify the number of cells.

The fluorescence data from the Victor reader were normalized to the cell number obtained from the Crystal Violet assay to account for variations in cell density. The normalized ROS levels were then analyzed to determine the impact of the EVOO treatments under physiological and ischemic conditions. This detailed protocol ensures accurate and reproducible ROS measurement in TNC WT cells, providing insights into the cellular oxidative stress response under different treatment conditions

#### 4.3.4. Vitality Assay

The vitality assay was conducted to assess the treated cell viability under both physiological and ischemic conditions. Initially, cells were seeded in quintuplicate into 96-well plates to ensure statistical robustness and an adequate sample size for analysis. The cells were then subjected to EVOO treatment for 24 h. This treatment was performed under two distinct conditions: physiological conditions and ischemic conditions, which involved 5 h of hypoxia followed by reoxygenation for the remaining 19 h.

After treatment, cells were fixed using a 4% paraformaldehyde solution (Thermo Fisher Scientific, Waltham, MA, USA). This fixation step was crucial to preserve cellular morphology and to prepare the cells for subsequent staining. Once the cells were adequately fixed, staining with Crystal Violet was conducted, a dye that binds to cellular DNA, thereby allowing for the visualization and quantification of viable cells.

Following the staining process, the cells were carefully washed with PBS to remove any excess Crystal Violet that did not bind to the cells. This washing step ensured that only the cells retaining the dye would contribute to measurements, thus providing an accurate cell viability representation.

Na-EtOH citrate solution (Sigma-Aldrich, Saint Louis, MO, USA) was introduced to the wells to quantify viable cells. This solution causes the stained cells to release the bound Crystal Violet dye into the solution, leading to cell discoloration. The extent of discoloration, which correlates with the number of viable cells, was then measured using a Victor plate reader. The Victor instrument provided precise absorbance readings, which were used to determine cell viability.

The Crystal Violet assay relies on the principle that viable cells remain attached to the well surface and retain the dye, whereas nonviable cells detach and do not retain the dye. Thus, the intensity of the color measured in the wells directly reflects the number of viable cells post-treatment. By comparing the absorbance values from different treatment conditions, the assay provides insights into the effects of EVOO treatment on cell viability under both normoxic and hypoxic conditions.

#### 4.3.5. XF Real-Time ATP Rate Assay

The XF Real-Time ATP Rate Assay was employed to quantify mitochondrial ATP production using Oligomycin, a specific inhibitor of mitochondrial ATP synthesis. Oligomycin injection leads to a reduction in the oxygen consumption rate (OCR), which, along with extracellular acidification rate (ECAR) data, is used to calculate the total proton efflux rate (PER). The assay also involves complete mitochondrial respiration inhibition using Rotenone and Antimycin A. This inhibition induces mitochondria-associated acidification, and combined with PER data, it enables the calculation of the glycolytic ATP (glycoATP) production rate (as detailed in Supplementary Material S3).

For the experimental procedure, cells were first plated into 24-well plates. After a 24-h treatment with EVOO under physiological and ischemic conditions, the treatment medium was replaced with DMEM adjusted to pH 7.4. The cells were then incubated in a 37 °C oven for 1 h for equilibration in the new medium.

Meanwhile, the Hydrate cartridge, which had been pre-incubated at 37 °C overnight with 500 μL of Calibration Solution (Agilent, Santa Clara, CA, USA), was prepared for use. The Sensor Cartridge ports were loaded with the necessary substrates: 1.5 μM Oligomycin to inhibit ATP synthesis and 0.5 μM Rotenone (Sigma-Aldrich, Saint Louis, MO, USA) combined with Antimycin A (Sigma-Aldrich, Saint Louis, MO, USA) to inhibit mitochondrial respiration.

After complete calibration, 24-well plates containing the cells were introduced into the assay platform, and the measurement was initiated. This setup allowed for the real-time monitoring of cellular respiration and glycolysis, providing detailed insights into the bioenergetic profiles of the cells under different treatment conditions. The data generated, including OCR and ECAR, were essential for calculating the total proton efflux rate and the glycolytic ATP production rate, thereby providing a comprehensive view of cellular energy metabolism.

#### 4.3.6. CO_2_/O_2_ Incubator

The investigation of the I/R model in astrocytes utilized a Sanyo O_2_/CO_2_ incubator, which facilitated precise control over the oxygen levels inside. This capability allowed for the simulation of ischemic conditions by adjusting the gas composition to 2% O_2_, 5% CO_2_, and 93% N_2_.

Astrocytes were placed in the O_2_/CO_2_ incubator for 5 h to induce ischemic conditions. Following this ischemic phase, two experimental protocols were followed: 1. Reoxygenation phase—after the initial 5 h of ischemia, cells were transferred back to a standard CO_2_ incubator with normal oxygen levels (referred to as reoxygenation) for an additional 19 h.

These conditions mimicked the astrocyte’s physiological responses to ischemic insult, allowing researchers to investigate cellular responses and adaptations under controlled ischemic and reperfusion conditions.

### 4.4. Statistics Analysis

GraphPad Prism version 6 (GraphPad Software Inc., La Jolla, CA, USA) was used for statistical analysis [75]. Data were initially subjected to a one-way Analysis Of Variance (ANOVA) to determine the overall significance among different treatment groups. The ANOVA test assesses whether there are any statistically significant differences between the means of the groups.

Following the ANOVA, post hoc comparisons were conducted using the Newman–Keuls test. This test was chosen to identify specific differences between pairs of groups while controlling for type I errors that can occur with multiple comparisons. The Newman–Keuls post hoc test sequentially compares the means of the groups and determines which specific pairs exhibit statistically significant differences.

Data are presented as mean ± standard deviation (SD) for each experimental group. A *p*-value < 0.05 was considered statistically significant for all analyses. This combination of ANOVA followed by the Newman-Keuls post hoc test ensured a robust data analysis, allowing for a detailed examination of the effects of different treatments under both physiological and ischemic conditions.

## 5. Conclusions

In conclusion, our study identified a protective effect of EVOO against cerebral ischemia in in vitro and in vivo models. The in vitro results suggest that EVOO treatment enhances cellular respiration and mitigates oxidative stress under hypoxic conditions. The in vivo experiments showed that EVOO treatment following recanalization one day after transient MCAo reduced infarct volumes and improved neurobehavioral deficits in rats. The protective mechanism is hypothesized to be mediated by PUFAs, which potentially safeguard the BBB, mitigate ROS production, and attenuate brain inflammation by preserving mitochondrial integrity and function. Ongoing translational investigations are needed to deeply explore the impact of transient MCAo and recanalization and synergistic interactions between EVOO compounds.

## Figures and Tables

**Table 1:** Fatty acid composition of olive oil.

Compound Name	Meas. R	Area %
Myristic	5.275	0.008
Palmitic	8.47	9.378
Palmitoleic 1	9.27	0.089
Palmitoleic 2	9.489	0.305
Heptadecenoic	11.956	0.046
Stearic	13.873	2.625
Oleic 2	15.00	75.649
Oleic 1	15.099	1.955
Linoleic	16.729	7.490
Linolenic	19.194	0.686
Arachic	20.089	0.476
Eicosenoic	20.886	0.447
Beenico	26.434	0.130
Erucic	29.415	0.034
Lignoceric	30.539	0.532

**Table 2:** Investigated polyphenol composition of olive oil.

Compound	Concentration (mg/g)
Kaempferol	6.77 ± 0.5
Acacetin	0.41 ± 0.15
Baicalein	0.4 ± 0.04
Diosmetin	0.51 ± 0.02
Chrysin	<LOQ *
(+/−)-Naringenin	0.18 ± 0.01
Baicalin	0.12 ± 0.01
Luteolin	+ **
Apigenin	<LOQ
Biochanin A	<LOQ
2,3-Dihydroxybenzoic acid	<LOQ
p-Coumaric acid	1.81 ± 0.08
Ferulic acid	0.06 ± 0.02
Phloretic acid	+
Catechol	0.75 ± 0.004
Salicylic acid	<LOQ
Vanillic acid	0.35 ± 0.04

* below the limit of detection. ** present but not quantifiable in the selected analytical condition.

**Table 3:** Optimized Q1 mass, production, and parameters for sMRM experiment of phenolic acids and flavonoids.

Metabolite	Metabolite Class	Precursor Ion (*m*/*z*)	Production (*m*/*z*)	DP ^a^	EP ^b^	CE ^c^	CXP ^d^	RT ^e^ (min)
Quinaldic acid	Internal Standard	174.05 [M+H]^+^	128.0	70	10	30	9	2.57
o-Anisic acid	Phenolic Acids	152.8 [M+H]^+^	135.1	70	10	17	20	4.78
2,3-Dihydroxybenzoic acid	Phenolic Acids	153.0 [M-H]^−^	109.0	−49	−10.9	−19	−5	2.94
Chlorogenic acid	Phenolic Acids	353.1 [M-H]^−^	190.8	−49	−10.9	−20	−10	3.99
Syringic acid	Phenolic Acids	197.0 [M-H]^−^	152.9	−49	−10.9	−16	−16	4.17
p-Coumaric acid	Phenolic Acids	163.0 [M-H]^−^	119.1	−49	−10.9	−12	−10	4.72
m-Hydrocoumaric acid	Phenolic Acids	165.0 [M-H]^−^	120.6	−49	−10.9	−18	−7	4.78
Ferulic acid	Phenolic Acids	193.1 [M-H]^−^	134.1	−49	−10.9	−20	−4	5.62
Sinapic acid	Phenolic Acids	223.1 [M-H]^−^	163.5	−49	−10.9	−19	−16	6.01
Aspirin	Phenolic Acids	179.1 [M-H]^−^	136.7	−49	−10.9	−12	−9	5.72
*trans*-Cinnamic acid	Phenolic Acids	147.0 [M-H]^−^	103.0	−49	−10.9	−14	−10	7.66
4-Hydroxybenzoic acid	Phenolic Acids	137.0 [M-H]^−^	92.7	−49	−10.9	−18	−7	1.84
2,6-Dihydroxybenzoic acid	Phenolic Acids	153.0 [M-H]^−^	108.7	−49	−10.9	−18	−19	6.44
Dihydrocaffeic acid	Phenolic Acids	181.1 [M-H]^−^	162.8	−49	−10.9	−19	−15	7.91
Caffeic acid	Phenolic Acids	179.0 [M-H]^−^	134.4	−49	−10.9	−20	−7.5	3.17
Phloretic acid	Phenolic Acids	165.1 [M-H]^−^	148.7	−49	−10.9	−20	−9	8.67
Hydroferulic acid	Phenolic Acids	195.1 [M-H]^−^	135.7	−49	−10.9	−15	−5.6	4.9
Ellagic acid dihydrate	Phenolic Acids	301.0 [M-H]^−^	144.8	−49	−10.9	−43	−17	7.1
5-Methoxysalicylic acid	Phenolic Acids	167.0 [M-H]^−^	107.6	−49	−10.9	−30	−14	6.6
Catechol	Phenolic Acids	109.0 [M-H]^−^	108.9	−49	−10.9	−11	−31	1.1
Gentisic acid	Phenolic Acids	153.0 [M-H]^−^	107.7	−49	−10.9	−27	−7	2.19
4-Acetocatechol	Phenolic Acids	151.0 [M-H]^−^	107.3	−49	−10.9	−27	−9	2.64
4-Methylcatechol	Phenolic Acids	123.0 [M-H]^−^	94.5	−49	−10.9	−22	−14	2.9
2,6-Dimethoxybenzoic acid	Phenolic Acids	181.1 [M-H]^−^	136.7	−49	−10.9	−10.6	−9	4.5
Acetylphloroglucinol	Phenolic Acids	167.0 [M-H]^−^	122.8	−49	−10.9	−23	−11	5.05
Salicylic acid	Phenolic Acids	137.0 [M-H]^−^	92.6	−49	−10.9	−25	−7	5.54
trans−2-Hydroxycinnamic acid	Phenolic Acids	163.0 [M-H]^−^	119.2	−49	−10.9	−15	−26	6.14
Caffeic acid dimethyl ether	Phenolic Acids	207.0 [M-H]^−^	102.7	−49	−10.9	−19	−16	7.34
3-Methoxyhydrocinnamic acid	Phenolic Acids	179.1 [M-H]^−^	119.9	−49	−10.9	−17.5	−13	7.48
Gallic acid	Phenolic Acids	169.0 [M-H]^−^	124.9	−49	−10.9	−21	−9	0.52
3,5-Dihydroxybenzoic acid	Phenolic Acids	153.0 [M-H]^−^	108.8	−49	−10.9	−15	−9	1.02
Vanillic acid	Phenolic Acids	167.0 [M-H]^−^	151.8	−49	−10.9	−18	−14	3.07
Nordihydroguaiaretic acid	Phenolic Acids	301.1 [M-H]^−^	121.6	−49	−10.9	−34.5	−23	8.25
Terephthalic acid	Phenolic Acids	165.0 [M-H]^−^	120.5	−49	−10.9	−14	−12	3.19
4-Acetylresorcinol	Phenolic Acids	151.0 [M-H]^−^	91.0	−49	−10.9	−25.5	−20	5.09
Rosmarinic acid	Phenolic Acids	359.1 [M-H]^−^	160.6	−49	−10.9	−16.5	−11	7.48
Caffeic acid phenethyl ester	Phenolic Acids	283.09 [M-H]^−^	134.8	−49	−10.9	−29	−29	8.35
2,3,4-Trihydroxybenzoic acid	Phenolic Acids	169.0 [M-H]^−^	150.8	−49	−10.9	−16.5	−16	1.15
2,4-Dihydroxybenzoic acid	Phenolic Acids	153.0 [M-H]^−^	109.1	−49	−10.9	−17	−8	2.66
3-Hydroxybenzoic acid	Phenolic Acids	137.0 [M-H]^−^	92.8	−49	−10.9	−17	−27	2.74
Coniferyl alcohol	Phenolic Acids	179.1 [M-H]^−^	145.6	−49	−10.9	−18	−5	4.5
m-Coumaric acid	Phenolic Acids	163.04 [M-H]^−^	118.8	−49	−10.9	−18	−19	5.38
2-Acetylresorcinol	Phenolic Acids	151.04 [M-H]^−^	134.6	−49	−10.9	−22	−7	5.96
3,4,5-Trimethoxycinnamic acid	Phenolic Acids	237.1 [M-H]^−^	103.2	−49	−10.9	−19	−27	7.61
Kaempferol	Flavonoids	285.0 [M-H]^−^	197.8	−56	−8.5	−36	−9	8.09
Phloretin	Flavonoids	273.1 [M-H]^−^	166.9	−56	−8.5	−24	−15	8
7,8-Dihydroxyflavone	Flavonoids	317.0 [M-H]^−^	178.8	−56	−8.5	−28	−19	7.48
Myricetin	Flavonoids	389.1 [M-H]^−^	148.8	−56	−8.5	−38	−31	8.5
Polydatin	Flavonoids	301.0 [M-H]^−^	150.3	−56	−8.5	−24	−5	7.88
Quercetin	Flavonoids	285.08 [M-H]^−^	184.5	−56	−8.5	−40	−13	8.09
Acacetin	Flavonoids	269.0 [M-H]^−^	194.6	−56	−8.5	−35	−34	8.21
Baicalein	Flavonoids	223.1 [M-H]^−^	91.6	−56	−8.5	−38	−8	8.33
4′-Hydroxychalcone	Flavonoids	289.1 [M-H]^−^	252.9	−56	−8.5	−19	−17	7.9
(+)-Catechin (hydrate)	Flavonoids	301.0 [M-H]^−^	150.3	−56	−8.5	−24	−5	7.88
Mangiferin	Flavonoids	421.1 [M-H]^−^	300.9	−56	−8.5	−27	−14	5.21
(+)-Taxifolin	Flavonoids	303.0 [M-H]^−^	285.0	−56	−8.5	−17	−20	5.97
Diosmetin	Flavonoids	299.0 [M-H]^−^	284.0	−56	−8.5	−28	−21	8.16
Morin	Flavonoids	301.0 [M-H]^−^	150.5	−56	−8.5	−31	−32	7.85
(−)-Epigallocatechin gallate hydrate	Flavonoids	457.1 [M-H]^−^	168.9	−56	−8.5	−16	−8	4.96
Chrysin	Flavonoids	253.1 [M-H]^−^	142.8	−56	−8.5	−41	−41	8.42
(+/−)-Naringenin	Flavonoids	271.1 [M-H]^−^	118.8	−56	−8.5	−28	−14	7.98
Baicalin	Flavonoids	445.1 [M-H]^−^	268.5	−56	−8.5	−27	−18	8.05
Resveratrol	Flavonoids	227.0 [M-H]^−^	184.4	−56	−8.5	−22	−9	7.34
Luteolin	Flavonoids	285.0 [M-H]^−^	133.0	−56	−8.5	−41	−37	7.3
Hesperidin	Flavonoids	609.2 [M-H]^−^	300.7	−56	−8.5	−31	−31	7.63
Fisetin	Flavonoids	285.0 [M-H]^−^	134.5	−56	−8.5	−27	−14	7.55
(−)-Epicatechin	Flavonoids	289.1 [M-H]^−^	252.8	−56	−8.5	−22	−22	7.75
Oxyresveratrol	Flavonoids	243.0 [M-H]^−^	200.3	−56	−8.5	−25	−6	6.64
Apigenin	Flavonoids	269.0 [M-H]^−^	116.4	−56	−8.5	−47	−7	8.1
*trans*-Pterostilbene	Flavonoids	255.1 [M-H]^−^	239.5	−56	−8.5	−27	−6	8.46
Rutin hydrate	Flavonoids	609.1 [M-H]^−^	299.7	−56	−8.5	−47	−9	7.27
Phloridzin	Flavonoids	435.1 [M-H]^−^	272.5	−56	−8.5	−20	−37	7.51
Daidzein	Flavonoids	253.1 [M-H]^−^	207.5	−56	−8.5	−35	−28	7.74
Hesperetin	Flavonoids	301.1 [M-H]^−^	163.4	−56	−8.5	−39	−11	8.05
Puerarin	Flavonoids	415.1 [M-H]^−^	294.9	−56	−8.5	−30	−12	5.09
Isoliquiritigenin	Flavonoids	255.1 [M-H]^−^	119.0	−56	−8.5	−27	−33	8.13
Piceatannol	Flavonoids	243.06 [M-H]^−^	158.3	−56	−8.5	−26	−13	6.35
Biochanin A	Flavonoids	283.1 [M-H]^−^	267.7	−56	−8.5	−26	−10	8.42
Formononetin	Flavonoids	267.1 [M-H]^−^	251.5	−56	−8.5	−28	−4	8.21
Diosmin	Flavonoids	607.2 [M-H]^−^	298.4	−56	−8.5	−22	−21	7.67
Naringin dihydrochalcone	Flavonoids	581.18 [M-H]^−^	273.0	−56	−8.5	−38	−10	7.75
Equol	Flavonoids	243.0 [M-H]^−^	200.8	−56	−8.5	−27	−19	6.33
Genistein	Flavonoids	269.0 [M-H]^−^	132.7	−56	−8.5	−34	−16	7.98

^a^ DP = declustering potential; ^b^ EP = entrance potential; ^c^ CE = collision energy; ^d^ CXP = collision cell exit potential; ^e^ RT= retention time.

**Table 4:** Linearity and sensitivity data.

Metabolite	Metabolite Class	Linearity Range (mg/mL)	r^2^	LOD	LOQ	Curve Equation
o-Anisic acid	Phenolic Acids	0.03–0.63	0.998	0.01	0.03	y = 3 × 10^6^x + 44,109
2,3-Dihydroxybenzoic acid	Phenolic Acids	0.03–5.00	0.997	0.006	0.03	y = 2 × 10^6^x + 189,509
Chlorogenic acid	Phenolic Acids	0.03–5.00	0.997	0.002	0.005	y = 1 × 10^6^x + 87,762
Syringic acid	Phenolic Acids	0.63–10.0	0.998	0.31	0.63	y = 17,964x − 1183.6
p-Coumaric acid	Phenolic Acids	0.03–2.50	0.998	0.005	0.02	y = 2 × 10^6^x + 12,2038
m-Hydrocoumaric acid	Phenolic Acids	0.31–10.0	0.999	0.03	0.31	y = 943,964x + 150,482
Ferulic acid	Phenolic Acids	0.03–10.0	0.999	0.002	0.03	y = 559,017x + 57,790
Sinapic acid	Phenolic Acids	0.03–10.0	0.999	0.002	0.005	y = 222,015x + 3754.9
Aspirin	Phenolic Acids	0.03–10.0	0.998	0.002	0.007	y = 405,391x – 67,347
*trans*-Cinnamic acid	Phenolic Acids	2.50–10.0	0.995	1.50	2.50	y = 36,651x – 21,342
4-Hydroxybenzoic acid	Phenolic Acids	0.03–1.25	0.998	0.006	0.03	y = 2 × 10^6^x + 26,546
2,6-Dihydroxybenzoic acid	Phenolic Acids	0.003–2.50	0.997	0.001	0.003	y = 4 × 10^6^x + 164,093
Dihydrocaffeic acid	Phenolic Acids	0.03–5.00	0.999	0.002	0.007	y = 538,677x + 27,979
Caffeic acid	Phenolic Acids	0.31–2.50	0.997	0.03	0.12	y = 1 × 10^6^x + 153,979
Phloretic acid	Phenolic Acids	Not quantifiable in the selected linearity range				
Hydroferulic acid	Phenolic Acids	0.03–5.00	0.999	0.001	0.003	y = 270,948x + 1777.7
Ellagic acid dihydrate	Phenolic Acids	0.03–1.25	0.997	0.007	0.03	y = 99,817x + 3393
5-Methoxysalicylic acid	Phenolic Acids	0.03–5.00	0.999	0.001	0.03	y = 3 × 10^6^x + 126,683
Catechol	Phenolic Acids	0.03–2.50	0.996	0.001	0.004	y = 1 × 10^6^x + 53,882
Gentisic acid	Phenolic Acids	0.003–1.25	0.999	0.001	0.003	y = 1 × 10^6^x + 6905.2
4-Acetocatechol	Phenolic Acids	0.03–2.50	0.996	0.007	0.03	y = 284,650x + 23,702
4-Methylcatechol	Phenolic Acids	0.31–10.0	0.999	0.03	0.14	y = 7528x + 825.75
2,6-Dimethoxybenzoic acid	Phenolic Acids	1.25–10.0	0.996	0.63	1.25	y = 49,710x + 38,212
Acetylphloroglucinol	Phenolic Acids	0.03–2.50	0.994	0.004	0.03	y = 2 × 10^6^x + 10,0214
Salicylic acid	Phenolic Acids	0.03–10.0	0.999	0.002	0.03	y = 3 × 10^6^x + 40,2246
trans-2-Hydroxycinnamic acid	Phenolic Acids	0.03–5.00	0.998	0.003	0.03	y = 2 × 10^6^x + 193,871
Caffeic acid dimethyl ether	Phenolic Acids	0.31–10.0	0.998	0.03	0.08	y = 42,692x − 4380
3-Methoxyhydrocinnamic acid	Phenolic Acids	0.31–10.0	0.997	0.03	0.11	y = 16,741x − 2051.4
Gallic acid	Phenolic Acids	0.03–2.50	0.999	0.005	0.03	y = 1 × 10^6^x + 37,728
3,5-Dihydroxybenzoic acid	Phenolic Acids	0.31–5.00	0.998	0.03	0.31	y = 695,154x + 108,182
Vanillic acid	Phenolic Acids	0.31–10.0	0.999	0.03	0.01	y = 25,878x − 747.51
Nordihydroguaiaretic acid	Phenolic Acids	0.03–2.50	0.999	0.001	0.002	y = 2 × 10^6^x + 21,094
Terephthalic acid	Phenolic Acids	0.03–2.50	0.999	0.01	0.03	y = 1 × 10^6^x + 35,990
4-Acetylresorcinol	Phenolic Acids	0.03–2.50	0.999	0.001	0.003	y = 906,372x + 17,474
Rosmarinic acid	Phenolic Acids	0.03–5.00	0.999	0.0005	0.001	y = 1 × 10^6^x − 3641.7
Caffeic acid phenethyl ester	Phenolic Acids	0.03–2.50	0.997	0.001	0.03	y = 5 × 10^6^x + 215,577
2,3,4-Trihydroxybenzoic acid	Phenolic Acids	0.03–0.60	0.999	0.001	0.003	y = 874,629x + 5590.4
2,4-Dihydroxybenzoic acid	Phenolic Acids	0.03–5.00	0.998	0.01	0.03	y = 928,863x + 55,270
3-Hydroxybenzoic acid	Phenolic Acids	0.31–5.00	0.998	0.03	0.31	y = 640,365x + 43,840
Coniferyl alcohol	Phenolic Acids	Not quantifiable in the selected linearity range				
m-Coumaric acid	Phenolic Acids	0.03–2.50	0.999	0.001	0.03	y = 2 × 10^6^x + 80,336
2-Acetylresorcinol	Phenolic Acids	0.03–1.25	0.996	0.002	0.005	y = 3× 10^6^ x + 99,822
3,4,5-Trimethoxycinnamic acid	Phenolic Acids	0.03–10.0	0.998	0.0003	0.03	y = 105,739x + 13,535
Kaempferol	Flavonoids	0.31–2.50	0.996	0.03	0.1	y = 31,889x + 313.64
Phloretin	Flavonoids	0.03–2.50	0.998	0.0004	0.001	y = 6 × 10^6^x + 181,590
Myricetin	Flavonoids	0.31–5.00	0.995	0.05	0.15	y = 669,096x – 127,523
Polydatin	Flavonoids	Not quantifiable in the selected linearity range				
Quercetin	Flavonoids	0.03–2.50	0.995	0.003	0.03	y = 232,809x + 10,141
Acacetin	Flavonoids	0.03–2.50	0.996	0.004	0.03	y = 141,397x + 7498.9
Baicalein	Flavonoids	0.03–5.00	0.998	0.004	0.03	y = 244,740x − 4360.9
4′-Hydroxychalcone	Flavonoids	0.03–2.50	0.995	0.002	0.03	y = 68,7940x + 15,160
(+)-Catechin (hydrate)	Flavonoids	0.31–1.25	0.997	0.03	0.06	y = 98,018x + 18,549
Mangiferin	Flavonoids	0.03–5.00	0.999	0.001	0.003	y = 1 × 10^6^x + 84,377
(+)-Taxifolin	Flavonoids	0.03–5.00	0.999	0.002	0.005	y = 2 × 10^6^x + 50,504
Diosmetin	Flavonoids	0.03–1.25	0.997	0.002	0.005	y = 6 × 10^6^x + 112,067
Morin	Flavonoids	0.03–5.00	0.997	0.002	0.005	y = 717,491x + 68,919
(−)-Epigallocatechin gallate hydrate	Flavonoids	0.03–10.0	0.998	0.008	0.03	y = 566,243x – 71,657
Chrysin	Flavonoids	0.03–5.00	0.996	0.0006	0.03	y = 598,355x + 56,081
(+/−)-Naringenin	Flavonoids	0.03–5.00	0.996	0.0005	0.001	y = 2× 10^6^ x − 15,413
Baicalin	Flavonoids	0.03–2.50	0.997	0.001	0.003	y = 1 × 10^6^x − 10,656
Resveratrol	Flavonoids	0.03–10.0	0.998	0.009	0.03	y = 46,605x + 1953.5
Luteolin	Flavonoids	Not quantifiable in the selected linearity range				
Hesperidin	Flavonoids	0.03–10.0	0.996	0.002	0.006	y = 433,221x + 1285.5
Fisetin	Flavonoids	0.03–5.00	0.996	0.002	0.03	y = 976,215x + 46,400
(−)-Epicatechin	Flavonoids	0.03–1.25	0.996	0.01	0.03	y = 141,591x + 674.48
Oxyresveratrol	Flavonoids	0.03–10.0	0.998	0.008	0.03	y = 30,531x + 1319
Apigenin	Flavonoids	0.03–1.25	0.994	0.001	0.005	y = 763,274x – 26,083
*trans*-Pterostilbene	Flavonoids	0.31–10.0	0.996	0.007	0.03	y = 529,98x − 1462.5
Rutin hydrate	Flavonoids	0.03–5.00	0.995	0.002	0.005	y = 1 × 10^6^x + 15,351
Phloridzin	Flavonoids	0.03–5.00	0.997	0.0005	0.002	y = 437,141x − 8944
Daidzein	Flavonoids	0.03–2.50	0.995	0.005	0.03	y = 247,718x + 22,978
Hesperetin	Flavonoids	0.03–5.00	0.996	0.0005	0.001	y = 619,584x – 14,267
Puerarin	Flavonoids	0.03–5.00	0.998	0.0005	0.002	y = 2 × 10^6^x − 1073
Isoliquiritigenin	Flavonoids	Not quantifiable in the selected linearity range				
Piceatannol	Flavonoids	0.03–10.0	0.997	0.009	0.03	y = 105,690x + 17,925
Biochanin A	Flavonoids	0.03–1.25	0.993	0.0004	0.001	y = 1 × 10^7^x + 287,361
Formononetin	Flavonoids	0.03–0.63	0.999	0.00001	0.003	y = 2 × 10^7^x + 33,838
Diosmin	Flavonoids	0.03–5.00	0.997	0.002	0.008	y = 221,995x − 1498.1
Naringin dihydrochalcone	Flavonoids	0.03–10.0	0.999	0.004	0.03	y = 418,240x + 42,251
Equol	Flavonoids	0.03–2.50	0.997	0.002	0.005	y = 727,655x + 19,681
Genistein	Flavonoids	0.03–5.00	0.994	0.01	0.03	y = 486,314x – 46,341

## Data Availability

The data to support the findings of this study will be available on request from the corresponding author.

## Supplementary Materials

The supporting information can be downloaded at https://www.mdpi.com/article/10.3390/ijms26041771/s1.
