# Supplementary material for: Bioactivity and Neuroprotective Effects of Extra Virgin Olive Oil in a Mouse Model of Cerebral Ischemia: An In Vitro and In Vivo Study
Source: Int J Mol Sci. 2025 Feb 19;26(4):1771. doi: 10.3390/ijms26041771 (PMC11855186; doi:10.3390/ijms26041771)
Supplement: Supplementary file 1 [file ijms-26-01771-s001.zip › ijms-3430416-supplementary/Summplentary Table S1.pdf]

Supplementary Table S1. Optimized Q1 mass, production, and parameters for the sMRM experiment of Phenolic Acids and Flavonoids.

Metabolite	Metabolite Class	Precursor ion (*m/z*)	Product ion (*m/z*)	DP[a]	EP[b]	CE[c]	CXP[d]	RT[e](min)
Quinaldic Acid	Internal Standard	174.05 $[M+H]^+$	128.0	70	10	30	9	2.57
o-Anisic Acid	Phenolic Acids	152.8 $[M+H]^+$	135.1	70	10	17	20	4.78
2,3-Dihydroxybenzoic acid	Phenolic Acids	153.0 $[M-H]^-$	109.0	-49	-10.9	-19	-5	2.94
Chlorogenic acid	Phenolic Acids	353.1 $[M-H]^-$	190.8	-49	-10.9	-20	-10	3.99
Syringic acid	Phenolic Acids	197.0 $[M-H]^-$	152.9	-49	-10.9	-16	-16	4.17
p-Coumaric acid	Phenolic Acids	163.0 $[M-H]^-$	119.1	-49	-10.9	-12	-10	4.72
m-Hydrocoumaric acid	Phenolic Acids	165.0 $[M-H]^-$	120.6	-49	-10.9	-18	-7	4.78
Ferulic acid	Phenolic Acids	193.1 $[M-H]^-$	134.1	-49	-10.9	-20	-4	5.62
Sinapic acid	Phenolic Acids	223.1 $[M-H]^-$	163.5	-49	-10.9	-19	-16	6.01
Aspirin	Phenolic Acids	179.1 $[M-H]^-$	136.7	-49	-10.9	-12	-9	5.72
trans-Cinnamic acid	Phenolic Acids	147.0 $[M-H]^-$	103.0	-49	-10.9	-14	-10	7.66
4-Hydroxybenzoic acid	Phenolic Acids	137.0 $[M-H]^-$	92.7	-49	-10.9	-18	-7	1.84
2,6-Dihydroxybenzoic acid	Phenolic Acids	153.0 $[M-H]^-$	108.7	-49	-10.9	-18	-19	6.44
Dihydrocaffeic acid	Phenolic Acids	181.1 $[M-H]^-$	162.8	-49	-10.9	-19	-15	7.91
Caffeic acid	Phenolic Acids	179.0 $[M-H]^-$	134.4	-49	-10.9	-20	-7.5	3.17
Phloretic acid	Phenolic Acids	165.1 $[M-H]^-$	148.7	-49	-10.9	-20	-9	8.67
Hydroferulic acid	Phenolic Acids	195.1 $[M-H]^-$	135.7	-49	-10.9	-15	-5.6	4.9
Ellagic acid dihydrate	Phenolic Acids	301.0 $[M-H]^-$	144.8	-49	-10.9	-43	-17	7.1
5-Methoxysalicylic acid	Phenolic Acids	167.0 $[M-H]^-$	107.6	-49	-10.9	-30	-14	6.6

Catechol	Phenolic Acids	109.0 $[M-H]^-$	108.9	-49	-10.9	-11	-31	1.1
Gentisic acid	Phenolic Acids	153.0 $[M-H]^-$	107.7	-49	-10.9	-27	-7	2.19
4-Acetocatechol	Phenolic Acids	151.0 $[M-H]^-$	107.3	-49	-10.9	-27	-9	2.64
4-Methylcatechol	Phenolic Acids	123.0 $[M-H]^-$	94.5	-49	-10.9	-22	-14	2.9
2,6-Dimethoxybenzoic acid	Phenolic Acids	181.1 $[M-H]^-$	136.7	-49	-10.9	-10.6	-9	4.5
Acetylphloroglucinol	Phenolic Acids	167.0 $[M-H]^-$	122.8	-49	-10.9	-23	-11	5.05
Salicylic acid	Phenolic Acids	137.0 $[M-H]^-$	92.6	-49	-10.9	-25	-7	5.54
trans-2-Hydroxycinnamic acid	Phenolic Acids	163.0 $[M-H]^-$	119.2	-49	-10.9	-15	-26	6.14
Caffeic acid dimethyl ether	Phenolic Acids	207.0 $[M-H]^-$	102.7	-49	-10.9	-19	-16	7.34
3-Methoxyhydrocinnamic acid	Phenolic Acids	179.1 $[M-H]^-$	119.9	-49	-10.9	-17.5	-13	7.48
Gallic acid	Phenolic Acids	169.0 $[M-H]^-$	124.9	-49	-10.9	-21	-9	0.52
3,5-Dihydroxybenzoic acid	Phenolic Acids	153.0 $[M-H]^-$	108.8	-49	-10.9	-15	-9	1.02
Vanillic acid	Phenolic Acids	167.0 $[M-H]^-$	151.8	-49	-10.9	-18	-14	3.07
Nordihydroguaiaretic Acid	Phenolic Acids	301.1 $[M-H]^-$	121.6	-49	-10.9	-34.5	-23	8.25
Terephthalic acid	Phenolic Acids	165.0 $[M-H]^-$	120.5	-49	-10.9	-14	-12	3.19
4-Acetylresorcinol	Phenolic Acids	151.0 $[M-H]^-$	91.0	-49	-10.9	-25.5	-20	5.09
Rosmarinic acid	Phenolic Acids	359.1 $[M-H]^-$	160.6	-49	-10.9	-16.5	-11	7.48
Caffeic acid phenethyl ester	Phenolic Acids	283.09 $[M-H]^-$	134.8	-49	-10.9	-29	-29	8.35
2,3,4-Trihydroxybenzoic acid	Phenolic Acids	169.0 $[M-H]^-$	150.8	-49	-10.9	-16.5	-16	1.15
2,4-Dihydroxybenzoic Acid	Phenolic Acids	153.0 $[M-H]^-$	109.1	-49	-10.9	-17	-8	2.66
3-Hydroxybenzoic acid	Phenolic Acids	137.0 $[M-H]^-$	92.8	-49	-10.9	-17	-27	2.74
Coniferyl Alcohol	Phenolic Acids	179.1 $[M-H]^-$	145.6	-49	-10.9	-18	-5	4.5
m-Coumaric acid	Phenolic Acids	163.04 $[M-H]^-$	118.8	-49	-10.9	-18	-19	5.38

2-Acetylresorcinol	Phenolic Acids	151.04 [M-H]$^-$	134.6	-49	-10.9	-22	-7	5.96
3,4,5-Trimethoxycinnamic acid	Phenolic Acids	237.1 [M-H]$^-$	103.2	-49	-10.9	-19	-27	7.61
Kaempferol	Flavonoids	285.0 [M-H]$^-$	197.8	-56	-8.5	-36	-9	8.09
Phloretin	Flavonoids	273.1 [M-H]$^-$	166.9	-56	-8.5	-24	-15	8
7,8-Dihydroxyflavone	Flavonoids	317.0 [M-H]$^-$	178.8	-56	-8.5	-28	-19	7.48
Myricetin	Flavonoids	389.1 [M-H]$^-$	148.8	-56	-8.5	-38	-31	8.5
Polydatin	Flavonoids	301.0 [M-H]$^-$	150.3	-56	-8.5	-24	-5	7.88
Quercetin	Flavonoids	285.08 [M-H]$^-$	184.5	-56	-8.5	-40	-13	8.09
Acacetin	Flavonoids	269.0 [M-H]$^-$	194.6	-56	-8.5	-35	-34	8.21
Baicalein	Flavonoids	223.1 [M-H]$^-$	91.6	-56	-8.5	-38	-8	8.33
4'-Hydroxychalcone	Flavonoids	289.1 [M-H]$^-$	252.9	-56	-8.5	-19	-17	7.9
(+)-Catechin (Hydrate)	Flavonoids	301.0 [M-H]$^-$	150.3	-56	-8.5	-24	-5	7.88
Mangiferin	Flavonoids	421.1 [M-H]$^-$	300.9	-56	-8.5	-27	-14	5.21
(+)-Taxifolin	Flavonoids	303.0 [M-H]$^-$	285.0	-56	-8.5	-17	-20	5.97
Diosmetin	Flavonoids	299.0 [M-H]$^-$	284.0	-56	-8.5	-28	-21	8.16
Morin	Flavonoids	301.0 [M-H]$^-$	150.5	-56	-8.5	-31	-32	7.85
(-)-Epigallocatechin gallate hydrate	Flavonoids	457.1 [M-H]$^-$	168.9	-56	-8.5	-16	-8	4.96
Chrysin	Flavonoids	253.1 [M-H]$^-$	142.8	-56	-8.5	-41	-41	8.42
(+/-)-Naringenin	Flavonoids	271.1 [M-H]$^-$	118.8	-56	-8.5	-28	-14	7.98
Baicalin	Flavonoids	445.1 [M-H]$^-$	268.5	-56	-8.5	-27	-18	8.05
Resveratrol	Flavonoids	227.0 [M-H]$^-$	184.4	-56	-8.5	-22	-9	7.34
Luteolin	Flavonoids	285.0 [M-H]$^-$	133.0	-56	-8.5	-41	-37	7.3

Hesperidin	Flavonoids	609.2 $[M-H]^-$	300.7	-56	-8.5	-31	-31	7.63
Fisetin	Flavonoids	285.0 $[M-H]^-$	134.5	-56	-8.5	-27	-14	7.55
(-)-Epicatechin	Flavonoids	289.1 $[M-H]^-$	252.8	-56	-8.5	-22	-22	7.75
Oxyresveratrol	Flavonoids	243.0 $[M-H]^-$	200.3	-56	-8.5	-25	-6	6.64
Apigenin	Flavonoids	269.0 $[M-H]^-$	116.4	-56	-8.5	-47	-7	8.1
trans-Pterostilbene	Flavonoids	255.1 $[M-H]^-$	239.5	-56	-8.5	-27	-6	8.46
Rutin Hydrate	Flavonoids	609.1 $[M-H]^-$	299.7	-56	-8.5	-47	-9	7.27
Phloridzin	Flavonoids	435.1 $[M-H]^-$	272.5	-56	-8.5	-20	-37	7.51
Daidzein	Flavonoids	253.1 $[M-H]^-$	207.5	-56	-8.5	-35	-28	7.74
Hesperetin	Flavonoids	301.1 $[M-H]^-$	163.4	-56	-8.5	-39	-11	8.05
Puerarin	Flavonoids	415.1 $[M-H]^-$	294.9	-56	-8.5	-30	-12	5.09
Isoliquiritigenin	Flavonoids	255.1 $[M-H]^-$	119.0	-56	-8.5	-27	-33	8.13
Piceatannol	Flavonoids	243.06 $[M-H]^-$	158.3	-56	-8.5	-26	-13	6.35
Biochanin A	Flavonoids	283.1 $[M-H]^-$	267.7	-56	-8.5	-26	-10	8.42
Formononetin	Flavonoids	267.1 $[M-H]^-$	251.5	-56	-8.5	-28	-4	8.21
Diosmin	Flavonoids	607.2 $[M-H]^-$	298.4	-56	-8.5	-22	-21	7.67
Naringin dihydrochalcone	Flavonoids	581.18 $[M-H]^-$	273.0	-56	-8.5	-38	-10	7.75
Equol	Flavonoids	243.0 $[M-H]^-$	200.8	-56	-8.5	-27	-19	6.33
Genistein	Flavonoids	269.0 $[M-H]^-$	132.7	-56	-8.5	-34	-16	7.98

[a]DP= declustering potential;EP= entrance potential [c]CE= collision energy; [d]CXP= Collision cell exit potential; [e]RT= retention time.

Supplementary Table 2. Linearity and sensitivity data.

Metabolite	Metabolite Class	Linearity range (μg/mL)	r^2	LOD	LOQ	Curve equation
o-Anisic Acid	Phenolic Acids	0.03–0.63	0.998	0.01	0.03	y = 3E+06x + 44109
2,3-Dihydroxybenzoic acid	Phenolic Acids	0.03–5.00	0.997	0.006	0.03	y = 2E+06x + 189509
Chlorogenic acid	Phenolic Acids	0.03–5.00	0.997	0.002	0.005	y = 1E+06x + 87762
Syringic acid	Phenolic Acids	0.63–10.0	0.998	0.31	0.63	y = 17964x - 1183.6
p-Coumaric acid	Phenolic Acids	0.03–2.50	0.998	0.005	0.02	y = 2E+06x + 122038
m-Hydrocoumaric acid	Phenolic Acids	0.31–10.0	0.999	0.03	0.31	y = 943964x + 150482
Ferulic acid	Phenolic Acids	0.03–10.0	0.999	0.002	0.03	y = 559017x + 57790
Sinapic acid	Phenolic Acids	0.03–10.0	0.999	0.002	0.005	y = 222015x + 3754.9
Aspirin	Phenolic Acids	0.03–10.0	0.998	0.002	0.007	y = 405391x - 67347
trans-Cinnamic acid	Phenolic Acids	2.50–10.0	0.995	1.50	2.50	y = 36651x - 21342
4-Hydroxybenzoic acid	Phenolic Acids	0.03–1.25	0.998	0.006	0.03	y = 2E+06x + 26546
2,6-Dihydroxybenzoic acid	Phenolic Acids	0.003–2.50	0.997	0.001	0.003	y = 4E+06x + 164093
Dihydrocaffeic acid	Phenolic Acids	0.03–5.00	0.999	0.002	0.007	y = 538677x + 27979
Caffeic acid	Phenolic Acids	0.31–2.50	0.997	0.03	0.12	y = 1E+06x + 153979
Phloretic acid	Phenolic Acids	Not quantifiable in the selected linearity range				
Hydroferulic acid	Phenolic Acids	0.03–5.00	0.999	0.001	0.003	y = 270948x + 1777.7
Ellagic acid dihydrate	Phenolic Acids	0.03–1.25	0.997	0.007	0.03	y = 99817x + 3393
5-Methoxysalicylic acid	Phenolic Acids	0.03–5.00	0.999	0.001	0.03	y = 3E+06x + 126683

Catechol	Phenolic Acids	0.03–2.50	0.996	0.001	0.004	y = 1E+06x + 53882
Gentisic acid	Phenolic Acids	0.003–1.25	0.999	0.001	0.003	y = 1E+06x + 6905.2
4-Acetocatechol	Phenolic Acids	0.03–2.50	0.996	0.007	0.03	y = 284650x + 23702
4-Methylcatechol	Phenolic Acids	0.31–10.0	0.999	0.03	0.14	y = 7528x + 825.75
2,6-Dimethoxybenzoic acid	Phenolic Acids	1.25 – 10.0	0.996	0.63	1.25	y = 49710x + 38212
Acetylphloroglucinol	Phenolic Acids	0.03–2.50	0.994	0.004	0.03	y = 2E+06x + 100214
Salicylic acid	Phenolic Acids	0.03–10.0	0.999	0.002	0.03	y = 3E+06x + 402246
trans-2-Hydroxycinnamic acid	Phenolic Acids	0.03–5.00	0.998	0.003	0.03	y = 2E+06x + 193871
Caffeic acid dimethyl ether	Phenolic Acids	0.31–10.0	0.998	0.03	0.08	y = 42692x - 4380
3-Methoxyhydrocinnamic acid	Phenolic Acids	0.31–10.0	0.997	0.03	0.11	y = 16741x - 2051.4
Gallic acid	Phenolic Acids	0.03–2.50	0.999	0.005	0.03	y = 1E+06x + 37728
3,5-Dihydroxybenzoic acid	Phenolic Acids	0.31–5.00	0.998	0.03	0.31	y = 695154x + 108182
Vanillic acid	Phenolic Acids	0.31–10.0	0.999	0.03	0.01	y = 25878x - 747.51
Nordihydroguaiaretic Acid	Phenolic Acids	0.03–2.50	0.999	0.001	0.002	y = 2E+06x + 21094
Terephthalic acid	Phenolic Acids	0.03–2.50	0.999	0.01	0.03	y = 1E+06x + 35990
4-Acetylresorcinol	Phenolic Acids	0.03–2.50	0.999	0.001	0.003	y = 906372x + 17474
Rosmarinic acid	Phenolic Acids	0.03–5.00	0.999	0.0005	0.001	y = 1E+06x - 3641.7
Caffeic acid phenethyl ester	Phenolic Acids	0.03–2.50	0.997	0.001	0.03	y = 5E+06x + 215577
2,3,4-Trihydroxybenzoic acid	Phenolic Acids	0.03–0.60	0.999	0.001	0.003	y = 874629x + 5590.4
2,4-Dihydroxybenzoic Acid	Phenolic Acids	0.03–5.00	0.998	0.01	0.03	y = 928863x + 55270
3-Hydroxybenzoic acid	Phenolic Acids	0.31–5.00	0.998	0.03	0.31	y = 640365x + 43840
Coniferyl Alcohol	Phenolic Acids	Not quantifiable in the selected linearity range				
m-Coumaric acid	Phenolic Acids	0.03–2.50	0.999	0.001	0.03	y = 2E+06x + 80336

2-Acetylresorcinol	Phenolic Acids	0.03–1.25	0.996	0.002	0.005	y = 3E+06x + 99822
3,4,5-Trimethoxycinnamic acid	Phenolic Acids	0.03–10.0	0.998	0.0003	0.03	y = 105739x + 13535
Kaempferol	Flavonoids	0.31–2.50	0.996	0.03	0.1	y = 31889x + 313.64
Phloretin	Flavonoids	0.03–2.50	0.998	0.0004	0.001	y = 6E+06x + 181590
Myricetin	Flavonoids	0.31–5.00	0.995	0.05	0.15	y = 669096x - 127523
Polydatin	Flavonoids	Not quantifiable in the selected linearity range				
Quercetin	Flavonoids	0.03–2.50	0.995	0.003	0.03	y = 232809x + 10141
Acacetin	Flavonoids	0.03–2.50	0.996	0.004	0.03	y = 141397x + 7498.9
Baicalein	Flavonoids	0.03–5.00	0.998	0.004	0.03	y = 244740x - 4360.9
4'-Hydroxychalcone	Flavonoids	0.03–2.50	0.995	0.002	0.03	y = 687940x + 15160
(+)-Catechin (Hydrate)	Flavonoids	0.31–1.25	0.997	0.03	0.06	y = 98018x + 18549
Mangiferin	Flavonoids	0.03–5.00	0.999	0.001	0.003	y = 1E+06x + 84377
(+)-Taxifolin	Flavonoids	0.03–5.00	0.999	0.002	0.005	y = 2E+06x + 50504
Diosmetin	Flavonoids	0.03–1.25	0.997	0.002	0.005	y = 6E+06x + 112067
Morin	Flavonoids	0.03–5.00	0.997	0.002	0.005	y = 717491x + 68919
(-)-Epigallocatechin gallate hydrate	Flavonoids	0.03–10.0	0.998	0.008	0.03	y = 566243x - 71657
Chrysin	Flavonoids	0.03–5.00	0.996	0.0006	0.03	y = 598355x + 56081
(+/-)-Naringenin	Flavonoids	0.03–5.00	0.996	0.0005	0.001	y = 2E+06x - 15413
Baicalin	Flavonoids	0.03–2.50	0.997	0.001	0.003	y = 1E+06x - 10656
Resveratrol	Flavonoids	0.03–10.0	0.998	0.009	0.03	y = 46605x + 1953.5
Luteolin	Flavonoids	Not quantifiable in the selected linearity range				
Hesperidin	Flavonoids	0.03–10.0	0.996	0.002	0.006	y = 433221x + 1285.5

Fisetin	Flavonoids	0.03–5.00	0.996	0.002	0.03	y = 976215x + 46400
(-)-Epicatechin	Flavonoids	0.03–1.25	0.996	0.01	0.03	y = 141591x + 674.48
Oxyresveratrol	Flavonoids	0.03–10.0	0.998	0.008	0.03	y = 30531x + 1319
Apigenin	Flavonoids	0.03–1.25	0.994	0.001	0.005	y = 763274x - 26083
trans-Pterostilbene	Flavonoids	0.31–10.0	0.996	0.007	0.03	y = 52998x - 1462.5
Rutin Hydrate	Flavonoids	0.03–5.00	0.995	0.002	0.005	y = 1E+06x + 15351
Phloridzin	Flavonoids	0.03–5.00	0.997	0.0005	0.002	y = 437141x - 8944
Daidzein	Flavonoids	0.03–2.50	0.995	0.005	0.03	y = 247718x + 22978
Hesperetin	Flavonoids	0.03–5.00	0.996	0.0005	0.001	y = 619584x - 14267
Puerarin	Flavonoids	0.03–5.00	0.998	0.0005	0.002	y = 2E+06x - 1073
Isoliquiritigenin	Flavonoids	Not quantifiable in the selected linearity range				
Piceatannol	Flavonoids	0.03–10.0	0.997	0.009	0.03	y = 105690x + 17925
Biochanin A	Flavonoids	0.03–1.25	0.993	0.0004	0.001	y = 1E+07x + 287361
Formononetin	Flavonoids	0.03–0.63	0.999	0.00001	0.003	y = 2E+07x + 33838
Diosmin	Flavonoids	0.03–5.00	0.997	0.002	0.008	y = 221995x - 1498.1
Naringin dihydrochalcone	Flavonoids	0.03–10.0	0.999	0.004	0.03	y = 418240x + 42251
Equol	Flavonoids	0.03–2.50	0.997	0.002	0.005	y = 727655x + 19681
Genistein	Flavonoids	0.03–5.00	0.994	0.01	0.03	y = 486314x - 46341
