# Supplementary material for: Bioactivity and Neuroprotective Effects of Extra Virgin Olive Oil in a Mouse Model of Cerebral Ischemia: An In Vitro and In Vivo Study
Source: Int J Mol Sci. 2025 Feb 19;26(4):1771. doi: 10.3390/ijms26041771 (PMC11855186; doi:10.3390/ijms26041771)
Supplement: Supplementary file 1 [file ijms-26-01771-s001.zip › ijms-3430416-supplementary/Supplementary Material S2 (2) (1).pdf]

**Stomach Tubing-Gavage**	Oral gavage, a method for administering a specific volume of an agent orally, was employed using the previously described dosage before surgery initiation. The animal's weight determined the appropriate dosage volume, and the gavage tube length was verified by measuring from the animal's head tip to the last rib. To prevent stomach perforation, especially in younger animals (<20 g), the tube was marked at the nose. Proper head placement involved gently extending it backward, and aligning the neck and esophagus. The gavage tube was carefully inserted through the mouth diastema and advanced along the upper palate, and the material was administered through a syringe attached to the tube's end. Following dosing, the tube was gently removed at the same insertion angle, and the animal was returned to the cage, and monitored for 5 to 10 min for signs of breathing difficulty or discomfort. Subsequently, animals were re-evaluated 12 and 24 h post-administration.

**Surgery**	The rats underwent inhalation anesthesia with isoflurane in an oxygen-introduced, compressed environment. Positioned in a supine orientation on the surgical table, the rat's snout was aligned with the stereotaxic apparatus opening, facilitating constant delivery of anesthetic (Fluriso-isoflurane, USP), oxygen, and compression through a calibrated vaporizer (maintained at 21°C). Surgical parameters were set at 3% for the vaporizer, 6 l/min for compressed air, and 300 cc/min for oxygen. After shaving neck hair and disinfecting the area, a midline incision exposed the omohyoid (OM), digastric (DM), and sternocleidomastoid (SCM) muscles. The carotid triangle was created by pulling the OM downward and the SCM laterally, revealing the right common carotid artery (CCA). The CCA was isolated and a clip was applied, followed by a cut in the occipital artery (OA) to visualize the internal carotid artery (ICA). The external carotid artery (ECA) was exposed, coagulated distally, and then cut. The distal ICA was visualized, allowing for potential clamping of the pterygopalatine artery (PPA). A clip was inserted into the ICA and CCA to ensure complete blood flow interruption. A hole was made in the ECA, and a 4-0 medium MCAO suture, with dimensions 0.19 mm diameter and 30 mm length (coated diameter 0.37 +/- 0.02 mm, coat length 2-3 mm down to the bifurcation), was inserted. After placing the suture in the ICA position, the ECA origin was sutured with 5-0 silk, the clip on the ICA was removed, and the suture was gently advanced until resistance was encountered. The procedure continued by removing the CCA clip, closing the skin with 3-0 silk, and ensuring a clean surgical site.

**Neurological Score Evaluation**	The neurological impairments were assessed through the blindly evaluated Modified Garcia score and beam walking test [26]. Rats were evaluated before surgery, after MCAO, and following recanalization. The modified Garcia score comprises six categories: spontaneous activity, symmetry in the movement of four limbs, forepaw outstretching, climbing, body proprioception, and response to vibrissae touch. Each category received a score ranging from 0 to 18. The beam walking test gauged the rat's coordination and ability to traverse a narrow wooden beam for 60 s, with scores from 0 to 4 (indicating not walking and falling, not walking and remaining on the beam, walking but falling, walking less than 20 cm, and walking beyond 20 cm), based on the time the rat fell, traveled, or stayed on the beam [27].
**Assessment of Cerebral Infarct Volume**	Triphenyl tetrazolium chloride (TTC) staining was exclusively employed to calculate infarct volume at 1 h, 90 min, and 2 h after MCAO in this study. Rats were anesthetized with isoflurane and then euthanized. The entire brain was promptly extracted and coronally sectioned into 2 mm thick slices using Stainless Steel Brain Matrices. Brain slices were incubated in 2% TTC solution (4g TTC in 200 ml of 1% Phosphate-buffered saline) for 10 min. Subsequently, the TTC solution was discarded, and PBS 1% was applied for 10 min before capturing photographs. The infarct area and the ipsilateral hemisphere of each brain slice were quantified using ImageJ (Laboratory for Optical and Computational Instrumentation (LOCI, University of Wisconsin, USA). The results were presented as a ratio of the corrected infarct volume to the entire contralateral hemispheric volume [28].

**Isolation Mitochondria from Rat's Brain**	The rat was euthanized, and the brain was promptly placed in an ice-cold isolation buffer. Subsequently, the brain was bifurcated into two hemispheres: the left ischemia side and the right control side. Both hemispheres were weighed, and placed in a beaker containing ice-cold Isolation medium A (Sucrose 320 mM Tris-HCl 10 mM; K+ EDTA 1 mM; BSA 2,5 g/l; Adjusted pH to 7.4 with Tris/HCl), and minced into pieces using scissors. The minced tissue was suspended with 5-10 volumes of ice-cold isolation medium A and transferred to a pre-cooled glass potter. After homogenizing the tissue with 10 strokes, the homogenate was transferred to a 50 mL falcon tube. It was then centrifuged at 1000 x g for 10 min at 4°C. The supernatant was transferred into a new tube and centrifuged at 5000 x g for 10 min at 4°C. The supernatant was discarded, and mitochondria (sediment) were re-suspended in isolation medium B (Without BSA). The suspension was centrifuged at 10,000 x g for 10 min at 4°C. After discarding the supernatant, mitochondria were re-suspended in 200 µl isolation medium B.
