# Supplementary material for: Bioactivity and Neuroprotective Effects of Extra Virgin Olive Oil in a Mouse Model of Cerebral Ischemia: An In Vitro and In Vivo Study
Source: Int J Mol Sci. 2025 Feb 19;26(4):1771. doi: 10.3390/ijms26041771 (PMC11855186; doi:10.3390/ijms26041771)
Supplement: Supplementary file 1 [file ijms-26-01771-s001.zip › ijms-3430416-supplementary/Supplementary Material S3 (1) (1).pdf]

**Clark Electrode and Polarographic Principle**	The polarographic technique enables the assessment of cellular oxygen consumption by measuring the current generated between two electrodes upon applying a voltage. The Hansatech model Clark oxygen electrode was utilized within a specialized electrochemical cell, featuring a platinum cathode and a silver anode immersed in an electrolyte solution. These electrodes were affixed to an epoxy resin disc, with the central cathode adhered to the resin in a domed structure, surrounded by a slit housing the circular silver anode. A semi-saturated KCl solution served as an electrolytic bridge, covering both the cathode and anode. The membrane preparation involved placing a sheet of spacer paper and a polytetrafluoroethylene (P.T.F.E) membrane onto the electrode, secured with a rubber ring (O-ring). The circular slit, housing the anode, also served as an electrolyte reservoir transported toward the cathode by the spacer paper. The P.T.F.E membrane, operating via reverse osmosis, selectively allowed the diffusion of oxygen from the sample chamber to the electrode compartment, preventing other substances from reaching the electrodes. An additional O-ring was placed externally, encasing the entire membrane and sealing the electrode at the sample chamber's base. This chamber was connected to a thermostatic bath maintaining a temperature of 37°C. An adjustable stopper sealed the chamber, preventing oxygen exchange with the external environment. A capillary in the stopper facilitated the removal of bubbles and the addition of reagents using Hamilton syringes. The oxygraph, mounted on a magnetic stirrer, facilitated constant sample agitation to keep cells in suspension and ensure even distribution of added substrates and inhibitors. Oxygen diffused from the sample chamber to the electrode

	compartment, and the application of a bias voltage of 700 mV between the two electrodes induced ionization of the electrolyte, resulting in current flow. As the electrolyte consumed oxygen, the oxygen tension at the cathode approached zero, promoting the diffusion of more oxygen across the membrane to compensate for the deficit. The resulting current was directly proportional to the oxygen concentration in the sample chamber, reflecting changes in dissolved oxygen and its consumption by the cellular mitochondrial respiratory chain. The Oxygraph Plus software, responsible for recording and digitizing current intensity, applied a calibration factor to process data in a cartesian graph, displaying nmol/ml of oxygen over time.

**Quantitative Assessment of Mitochondrial Respiratory Fluxes**	Before sample analysis, the instrument underwent calibration by introducing 1 ml of double-distilled water into the oxygraph chamber. Upon achieving signal stability, sodium dithionite was added, and the chamber was sealed with the stopper. After reaching stability, indicated by an ideal calibration factor of 0.1, the chamber underwent a thorough rinsing with double-distilled water to eliminate the dithionite, and 400 µl of double-distilled water was introduced. Upon stabilization of oxygen concentration around 217 µM (standard value at 37 °C and atmospheric pressure), the water was replaced with 830 µl of TD reaction buffer (0.137 M NaCl, 5 mM KCl, Na2HPO4, 0.7 mM, 25 mM Tris/Cl, pH 7.4). Following stability, the instrument was prepared for measuring cellular oxygen consumption. Approximately 100 µl of TD reaction buffer was withdrawn from the chamber to resuspend the pellet obtained from detached cells. The cell suspension was then transferred to the thermostated oxygraph chamber set at 37°C with magnetic stirring (speed 80). Subsequently, 30 µl was withdrawn, with 10 µl allocated for cell counting, 20 µl for protein measurement, and the remaining 800 µl reserved for respirometric analysis. The analysis aimed to measure oxygen consumption attributed to the activity of each complex in the mitochondrial respiratory chain. Specific substrates and inhibitors for each complex were sequentially added to the cell suspension at regular intervals of approximately 2 min to allow for respiration stabilization and enhance measurement reliability.

**Calculation of Glycolytic ATP Production Rate**	In the glycolytic pathway, the conversion of one molecule of glucose to lactate produces two molecules each of ATP, H+, and lactate. The rate of ATP production in the glycolytic pathway (glycoATP Production Rate) is equivalent to Glycolytic Proton Efflux Rate (glycoPER). It is calculated using the validated approach from the Agilent Seahorse XF Glycolytic Rate Assay. Calculation of Mitochondrial ATP production rate: The rate of oxygen consumption coupled to ATP production during OXPHOS is calculated as the OCR inhibited by the ATP synthase inhibitor, oligomycin. Transformation of OCRATP to the rate of mitochondrial ATP production involves multiplying by 2 to convert molecules of O2 to oxygen atoms consumed. This is then multiplied by the P/O ratio, representing the number of molecules of ADP phosphorylated to ATP per atom of O reduced by an electron pair flowing through the electron transfer chain. The total cellular ATP production rate is the sum of glycolytic and mitochondrial ATP production rates.
